# Supplementary material for: Feeling moved by music: Investigating continuous ratings and acoustic correlates
Source: PLoS One. 2022 Jan 12;17(1):e0261151. doi: 10.1371/journal.pone.0261151 (PMC8754323; doi:10.1371/journal.pone.0261151)
Supplement: S1 File — Supplementary information, including S1–S9 Tables and S1–S9 Figs. (DOCX) [file pone.0261151.s001.docx]

# Feeling moved by music: Investigating continuous ratings and acoustic correlates

(Vuoskoski, Zickfeld, Alluri, Moorthigari & Seibt)

# Supplementary Material

## **1. Pilot Study**

- 1. Methods

In total 35 US participants were recruited on Amazon MTurk (14 females, 21 males) ranging from 22 to 59 years of age (*M* = 35.57, *SD* = 9.76). Each participant was presented with six different musical pieces from a total pool of 12 songs; 16 participants listened to the first six songs and 19 participants to the other half (Table S1). Some musical pieces were chosen based on previous studies (Eerola et al., 2016; Silvia et al., 2015; Vuoskoski & Eerola, 2017), while others were selected because they were assumed to *move* listeners. An overview of all songs is presented in Table S1. Each song was cut after four minutes to allow the same length across pieces. After each song, participants were presented with the same ratings.

Table S1. Overview of musical pieces and enjoyment and familiarity ratings.

| Song Code | Artist/Composer | Song Name | *n* | Enjoyment (Liking) | Familiarity |
| --- | --- | --- | --- | --- | --- |
| *Grieg* | Edvard Grieg | Morning Mood | 16 | 4.5 (1.41) | .69 (1.20) |
| *Ave Maria* | Yo-Yo Ma, Kathryn Stott/J. S. Bach | Ave Maria | 16 | 4 (1.86) | 3.12 (1.31) |
| *Vltava* | Berlin Philharmonics/  Smetana | Vltava | 16 | 4 (1.79) | 3.12 (1.26) |
| *Band of Brothers* | Michael Kamen | Suite Two (Band of Brothers) | 16 | 4.06 (1.69) | 3.31 (1.01) |
| *Nils Frahm* | Nils Frahm | Re (Helios Rework) | 16 | 3.88 (2.06) | 3.06 (1.34) |
| *Olafur Arnalds* | Ólafur Arnalds | þÚ Ert Sólin | 16 | 3.38 (1.86) | 3.44 (1.21) |
| *Schubert/Liszt* | F. Schubert, F. Liszt | Serenade | 19 | 3.63 (1.74) | 3.26 (.99) |
| *Explosions* | Explosions in the Sky | So Long, Lonesome | 19 | 4 (1.63) | 3.47 (.70) |
| *Sigur Ros* | Sigur Ros | Hoppipolla | 19 | 4.21 (1.93) | 3.16 (1.26) |
| *Oblivion* | Piazzolla/Hauser | Oblivion | 19 | 4.32 (1.34) | 3.37 (.83) |
| *Rachmaninov* | Rachmaninov/Luka Sulic | Vocalize | 19 | 3.63 (1.21) | 3.32 (1.00) |
| *Allegri* | Allegri | Miserere mei, deus | 19 | 3.79 (1.72) | 3.05 (1.22) |

Note. Enjoyment and familiarity rated on 7-point scales. Higher responses indicate higher enjoyment, while lower responses indicate higher familiarity.

After being presented with informed consent, participants were first asked to indicate states they experienced while listening to the musical piece. They were asked to indicate how *moved, sad, touched* they felt and to what degree they felt *a sense of connectedness*. Afterwards, we asked about physiological reactions including *moist eyes, tears, goosebumps, chills or shivers,* and *a feeling in the chest*. Then perceived attributions were indicated for *beautiful, intense, sad, joyful* and participants rated the degree the piece expressed *loneliness/separation* and *connectedness/closeness*. Finally, participants rated how much they liked the song and how familiar they were with the piece. All ratings were completed on 7-point scales ranging from *0 (not at all)* to *6 (very much)*. For the familiarity item low ratings indicated high familiarity *(definitely yes)* and high ratings low familiarity *(definitely not)*.

1.2 Results

First, we averaged ratings of felt *moved* and *touched* into a *feeling moved/touched* composite score as done in previous studies (*r_(210)_* = .80; Seibt et al., 2017). In order to explore associations among the ratings, we calculated correlations among the different items (Figure S1).


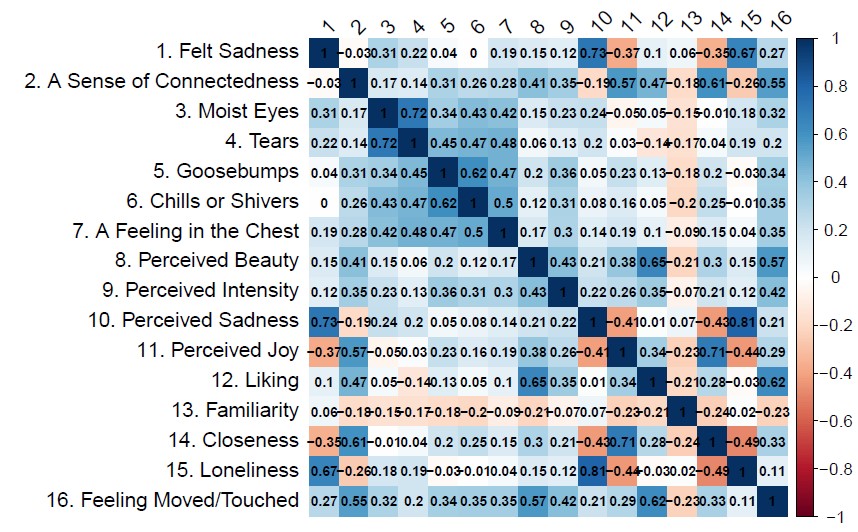
Figure S1. Pearson correlations among the main ratings (*n* = 210).

Ratings of feeling moved and touched correlated positively with experiencing a sense of connectedness (*r* = .55), as well as perceived beauty (*r* = .57) and enjoyment (*r* = .62) replicating previous studies (Vuoskoski & Eerola, 2017).

In order to split songs into categories of *sadly moving* and *joyfully moving* pieces, we first explored to what degree songs were perceived as sad, joyful and experienced as moving and touching (Figure S2).


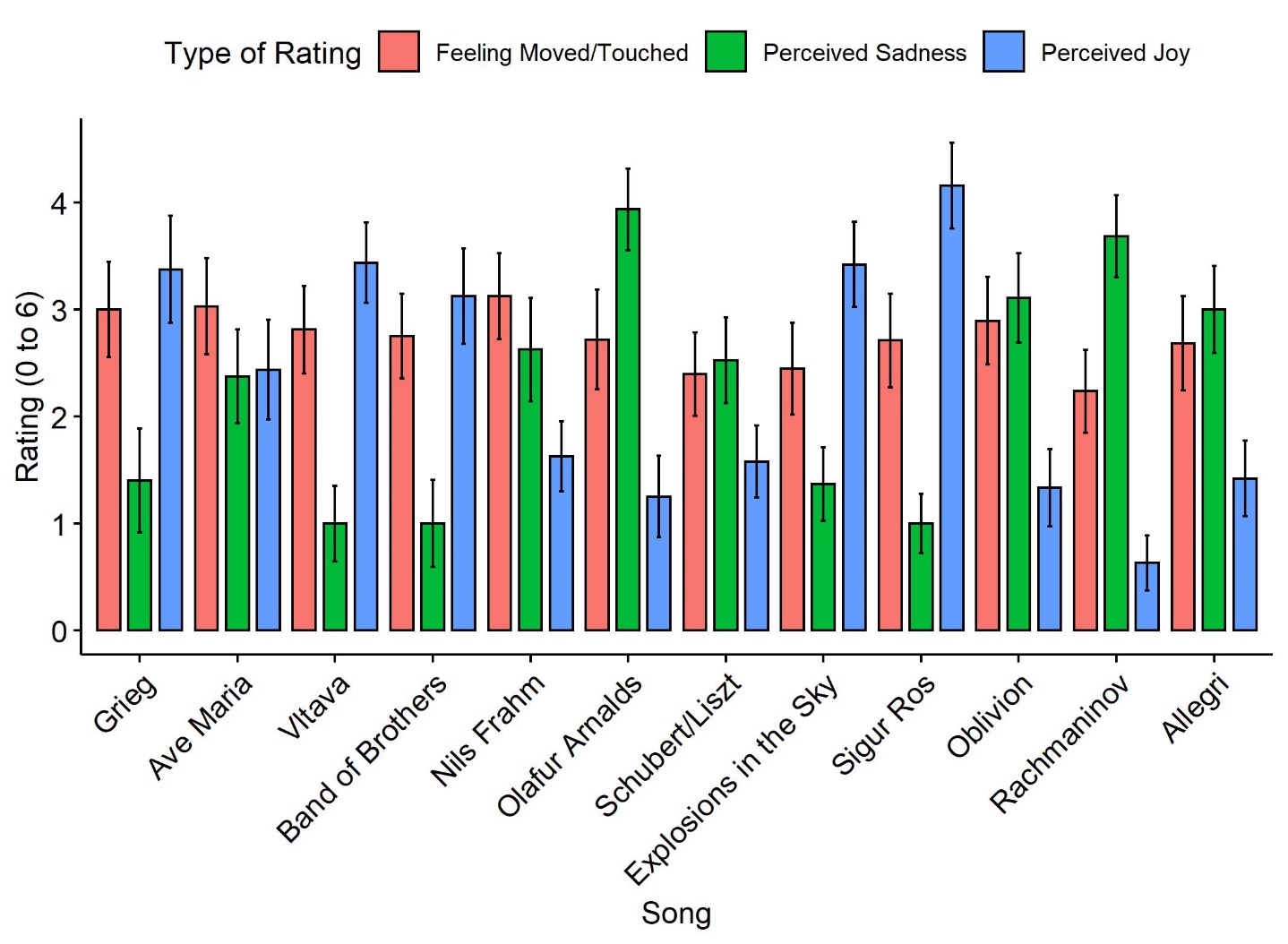
Figure S2. Ratings of feeling moved/touched, perceived sadness, and perceived joy by song.

In general, all songs received similar ratings on feeling moved/touched. *Nils Frahm* evoked the strongest ratings of feeling moved/touched, while this was lowest for Rachmaninov. We identified *Olafur Arnalds, Oblivion, Rachmaninov, Allegri, Nils Frahm,* and *Ave Maria* as the rated highest on perceived sadness. On the other hand, *Sigur Ros, Grieg, Vltava, Band of Brothers*, and *Explosions* were identified as being highest on perceived joy. Further, we employed multidimensional scaling to identify clusters among the songs focusing on the ratings of feeling moved, feeling touched, perceived sadness, and perceived joy. The final plot is presented in Figure S3.

The two dimensions were best identified as *valence* and the degree of *movingness*. The two clusters identified could be best summarized as sad and joyful themes, with sadder songs showed higher variation in the degree of valence. For each type we chose three different songs based on several criteria: first, the needed to show a considerable amount on the respective valence dimension; second, we opted for some variation in the degree of movingness not only selecting the most moving pieces; third, to control for the degree of familiarity we focused on musical pieces that were rated low on familiarity; fourth, we attempted to include musical pieces featuring a variation of instrumentation (i.e. not only solo piano pieces but also pieces with vocals or orchestral instrumentation). For the *joyfully moving* songs we selected *Sigur Ros*, *Vltava,* and *Band of Brothers*. *Grieg* was not chosen as it was rated with high familiarity by most participants. For the *sadly moving* songs we selected *Oblivion*, *Olafur Arnalds*, and *Allegri*. In addition, we selected *Explosions* as a *neutral* control category. However, this decision was originally based on a coding error as codings for *Ave Maria*, rated to a similar degree as sad and joyful, were confused with *Explosions*.


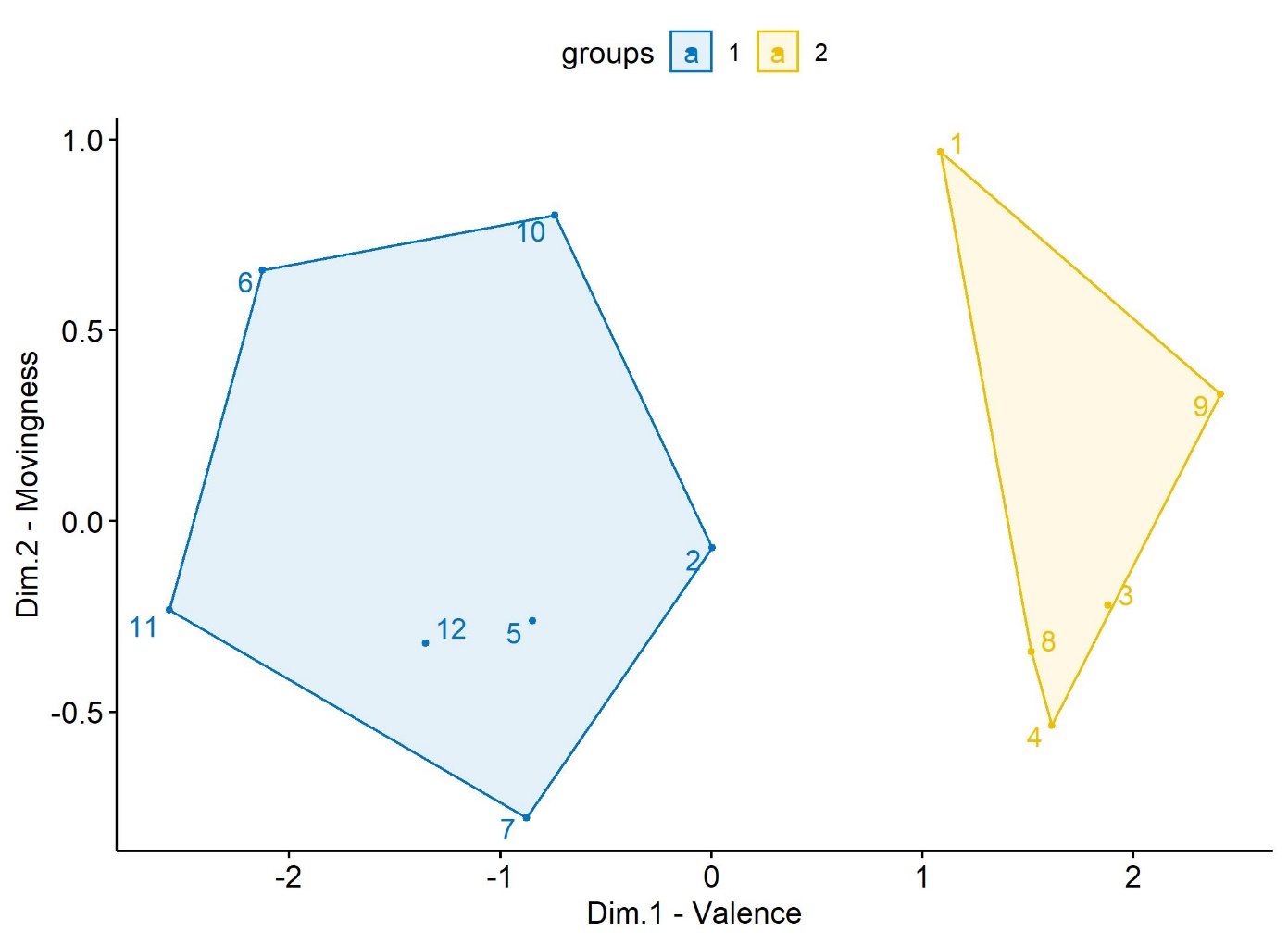


Figure S3. Scatter plot showing multidimensional scaling arrangement using 2 clusters. 1 = Grieg, 2 = Ave Maria, 3 = Vltava, 4 = Band of Brothers, 5 = Nils Frahm, 6 = Olafur Arnalds, 7 = Schubert/Liszt, 8 = Explosions, 9 = Sigur Ros, 10 = Oblivion, 11 = Rachmaninov, 12 = Allegri.

## **Main Study**


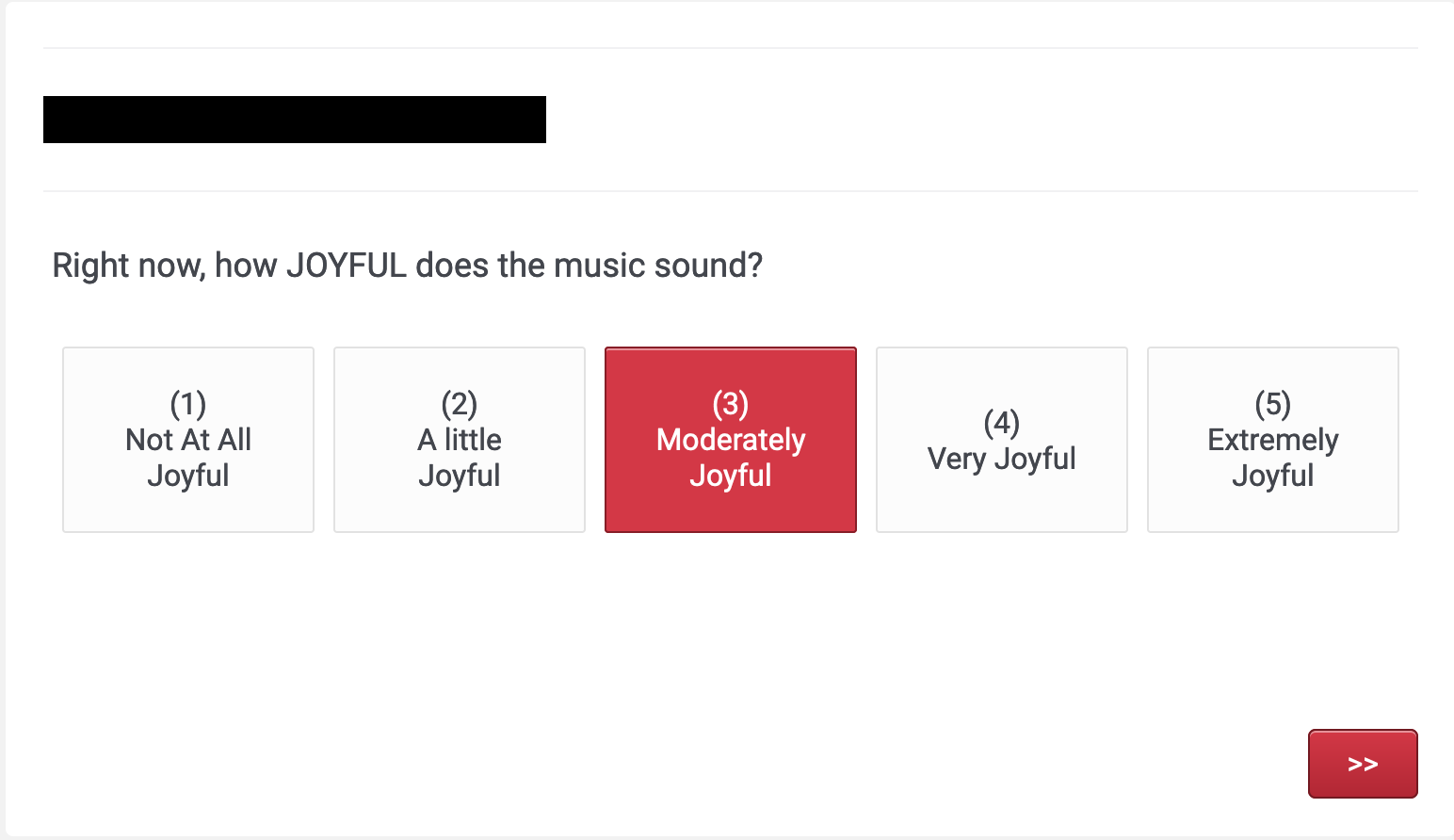


Figure S4. Example screenshot of continuous self-report paradigm. The scale point in red represents the one selected by the participant. Participants were able to change their rating by using their mouse, their arrow keys to navigate in an ascending or descending order, or use their number keys to select a specific response.

2.1 Sample Size Justification.

We performed a post-hoc precision analysis based on McKeown & Sneddon (2013) to justify our sample size decision. We used generalized additive models (GAM) in *mgcv* (Wood, 2001) and focused on the effect size R^2^. We employed a subsampling approach and focused on the musical excerpt x rating scale combination with the largest cell size (which was the connectedness rating for Explosions with *n* = 71, see Table S4). Following McKeown & Sneddon (2013), we assumed that the obtained R^2^ represents the *population* effect size. We then randomly sampled subsamples including between 2 and 70 participants. For each sample size, 1000 samples were picked randomly. For each subsample we computed a GAM with the connectedness rating as the outcome and time as the predictor, applying a cubic spline smoothing similar to the transformation in the main manuscript. The final distribution of effect sizes and their 95% bootstrapped confidence intervals is provided in Figure S5.1. A possible limitation of this approach is the fact that the selected cell had a rather high ICC and thereby high overlap among individual raters (see Table S4). Therefore, we repeated the same procedure for a different cell that showed a low ICC. We picked the Vlatava perceived sadness combination, as it showed the second lowest ICC, but still had a high enough cell size to make the simulation feasible (*n* = 50). An overview is presented in Figure S5.2. Both simulations show that cell sizes between 40-50 are sufficient to precisely estimate the *population* effect size.


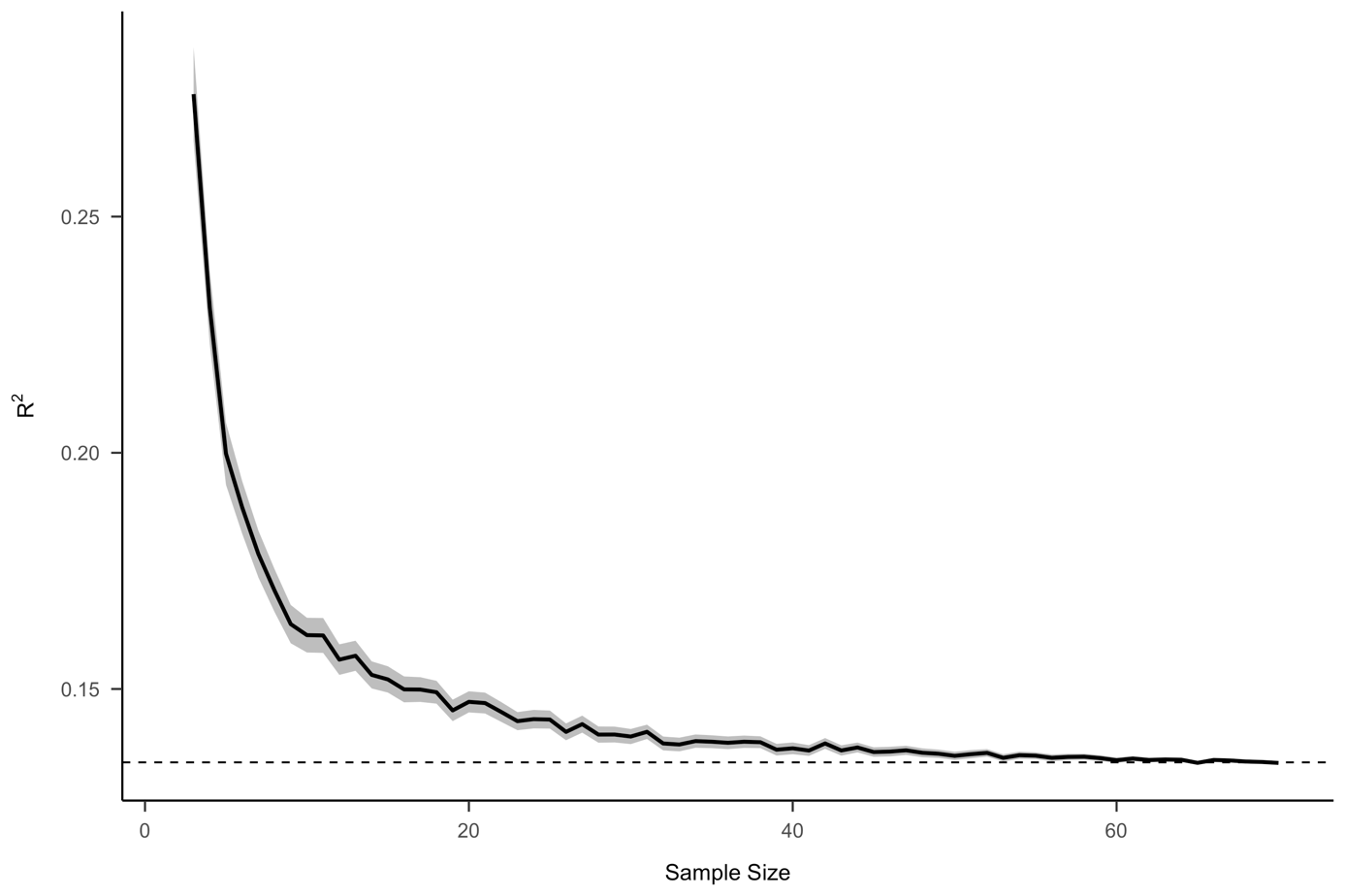


Figure S5.1. Randomly sampled effect sizes for GAMs of different sample sizes focusing on the connectedness ratings for Explosions. Grey shaded area represents 95% confidence intervals.


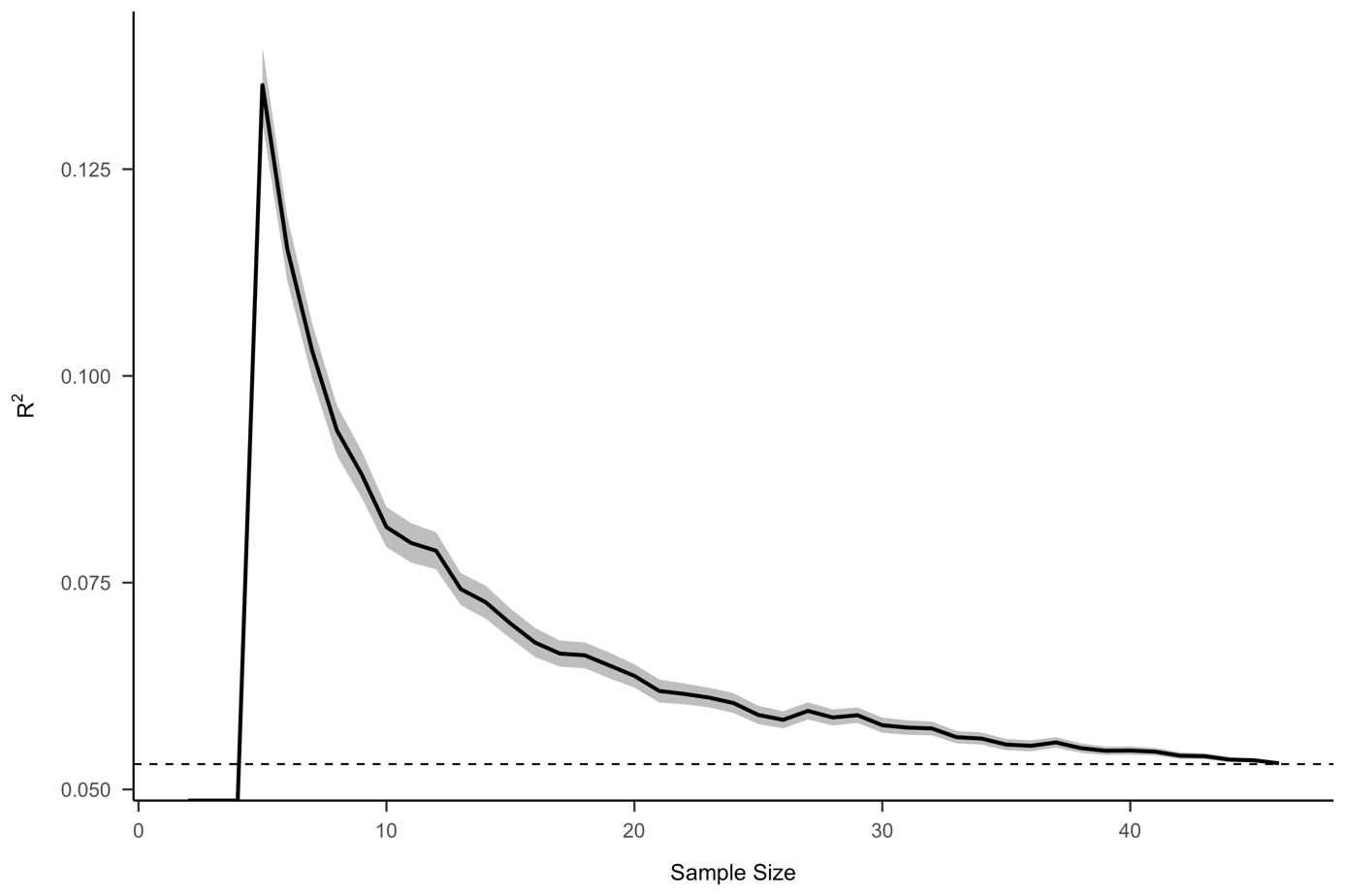


Figure S5.2. Randomly sampled effect sizes for GAMs of different sample sizes focusing on the perceived sadness ratings for Vltava. Grey shaded area represents 95% confidence intervals.

2.2 Duration of Chill Responses

We also explored the duration of chill responses. Based on our rating scale we considered everything above the rating of 1 (not at all) as a chill response. In total, participants indicated experiencing some kind of chills responses in 54.89% of all cases and no chill responses in 45.11%. We then considered the different ratings between 2 and 5 for the chill scale. Average time of chill responses ranged between 15.62 (4 Very Much) and 21.20s (2 A Little Chills). Overall, the average chill response for some type of chills (between 2 and 5) was 60.72s (ranging from 1 to 176s).

Table S2. Overview of musical pieces and enjoyment and familiarity ratings.

| Song Code | Artist/Composer | Song Name | Length (*s*) | *n* | Enjoyment (Liking) | Familiarity | | Tears | |
| --- | --- | --- | --- | --- | --- | --- | --- | --- | --- |
|  | *Sadly Moving* | | | | | |  | |  |
| *Allegri* | Allegri | Miserere mei, deus | 136 | 352 | 3.15 (1.86) | 3.39 (.97) | | .44 (1.02) | |
| *Olafur* | Ólafur Arnalds | þÚ Ert Sólin | 151 | 369 | 3.35 (1.70) | 3.67 (.67) | | .51 (1.10) | |
| *Oblivion* | Piazzolla/Hauser | Oblivion | 133 | 368 | 3.52 (1.73) | 3.48 (.87) | | .47 (1.08) | |
|  |  |  |  |  |  |  | |  | |
|  | *Joyfully Moving* | | | | | |  | |  |
| *Explosions* | Explosions in the Sky | So Long, Lonesome | 169 | 362 | 3.96 (1.54) | 3.58 (.92) | | .53 (1.19) | |
| *Vltava* | Berlin Philharmonics/  Smetana | Vltava | 180 | 370 | 3.74 (1.54) | 3.15 (1.20) | | .31 (.92) | |
| *Hoppipolla* | Sigur Ros | Hoppipolla | 115 | 264 | 3.62 (1.68) | 3.36 (1.12) | | .39 (.98) | |
| *Band of Brothers* | Michael Kamen | Suite Two (Band of Brothers soundtrack) | 162 | 360 | 3.96 (1.60) | 3.34 (1.00) | | .41 (1.08) | |

Note. Enjoyment and familiarity rated on 7-point scales. Higher responses indicate higher enjoyment, while lower responses indicate higher familiarity. For all stimuli see: https://youtube.com/playlist?list=PLc8nlHQh9a0GQB3dfDP2cRLhlq21YJieo

Table S3. Musical pieces average ratings over each participant and time series for the seven ratings separately.

|  | Sadness | Joy | Touched/  Moved | Beauty | Connectedness | Warmth | Chills |
| --- | --- | --- | --- | --- | --- | --- | --- |
| Song Code | *M (SD)* | | | | | | |
| *Sadly Moving* | | | | | | | |
| *Allegri* | 2.59 (1.13) | 1.57 (.82) | 2.24 (1.12) | 2.58 (1.18) | 1.92 (1.08) | 2.00 (1.11) | 1.91 (1.01) |
| *Olafur* | 2.75 (1.15) | 1.73 (0.86) | 2.46 (1.14) | 2.88 (1.21) | 2.68 (1.25) | 2.2 (1.15) | 1.87 (1.08) |
| *Oblivion* | 3.00 (1.19) | 1.81 (1.00) | 2.51 (1.23) | 3.28 (1.33) | 2.53 (1.24) | 2.26 (1.14) | 2.06 (1.04) |
| *Joyfully Moving* | | | | | | | |
| *Explosions* | 2.18 (1.04) | 2.86 (1.23) | 2.8 (1.33) | 3.08 (1.29) | 2.74 (1.33) | 2.79 (1.33) | 2.00 (1.04) |
| *Vltava* | 1.55 (0.78) | 2.99 (1.18) | 2.82 (1.13) | 3.06 (1.26) | 2.7 (1.28) | 2.34 (1.25) | 1.94 (1.07) |
| *Hoppipolla* | 1.53 (.74) | 3.27 (1.37) | 2.54 (1.34) | 3.25 (1.28) | 2.63 (1.32) | 2.66 (1.30) | 1.98 (1.12) |
| *Brothers* | 1.69 (0.92) | 3.32 (1.25) | 2.58 (1.21) | 3.26 (1.21) | 2.78 (1.26) | 2.47 (1.23) | 1.99 (1.24) |

Table S4. Intraclass correlation coefficients (and their 95% CIs) for each song excerpt and scale separately.

| Song | Scale | ICC | CI LB | CI UB | n |
| --- | --- | --- | --- | --- | --- |
| 1 | 1 | 0.88 | 0.84 | 0.9 | 58 |
| 1 | 2 | 0.75 | 0.69 | 0.8 | 45 |
| 1 | 3 | 0.86 | 0.82 | 0.89 | 52 |
| 1 | 4 | 0.88 | 0.84 | 0.9 | 53 |
| 1 | 5 | 0.84 | 0.8 | 0.88 | 60 |
| 1 | 6 | 0.83 | 0.77 | 0.87 | 39 |
| 1 | 7 | 0.78 | 0.72 | 0.83 | 48 |
| 2 | 1 | 0.86 | 0.83 | 0.89 | 60 |
| 2 | 2 | 0.88 | 0.85 | 0.91 | 50 |
| 2 | 3 | 0.92 | 0.9 | 0.94 | 54 |
| 2 | 4 | 0.92 | 0.9 | 0.94 | 60 |
| 2 | 5 | 0.9 | 0.86 | 0.92 | 40 |
| 2 | 6 | 0.86 | 0.82 | 0.89 | 58 |
| 2 | 7 | 0.86 | 0.83 | 0.89 | 51 |
| 3 | 1 | 0.89 | 0.86 | 0.92 | 62 |
| 3 | 2 | 0.89 | 0.87 | 0.92 | 59 |
| 3 | 3 | 0.87 | 0.83 | 0.9 | 55 |
| 3 | 4 | 0.84 | 0.79 | 0.89 | 32 |
| 3 | 5 | 0.86 | 0.82 | 0.9 | 52 |
| 3 | 6 | 0.92 | 0.9 | 0.94 | 64 |
| 3 | 7 | 0.85 | 0.81 | 0.88 | 48 |
| 4 | 1 | 0.76 | 0.71 | 0.81 | 40 |
| 4 | 2 | 0.95 | 0.94 | 0.96 | 52 |
| 4 | 3 | 0.92 | 0.89 | 0.94 | 40 |
| 4 | 4 | 0.91 | 0.89 | 0.93 | 62 |
| 4 | 5 | 0.92 | 0.89 | 0.93 | 71 |
| 4 | 6 | 0.9 | 0.87 | 0.93 | 48 |
| 4 | 7 | 0.91 | 0.88 | 0.93 | 52 |
| 5 | 1 | 0.71 | 0.65 | 0.76 | 47 |
| 5 | 2 | 0.94 | 0.93 | 0.95 | 56 |
| 5 | 3 | 0.94 | 0.93 | 0.96 | 65 |
| 5 | 4 | 0.88 | 0.85 | 0.91 | 52 |
| 5 | 5 | 0.88 | 0.85 | 0.91 | 51 |
| 5 | 6 | 0.87 | 0.84 | 0.9 | 54 |
| 5 | 7 | 0.82 | 0.77 | 0.86 | 48 |
| 6 | 1 | 0.36 | 0.25 | 0.47 | 32 |
| 6 | 2 | 0.93 | 0.9 | 0.95 | 35 |
| 6 | 3 | 0.92 | 0.89 | 0.94 | 43 |
| 6 | 4 | 0.92 | 0.9 | 0.94 | 41 |
| 6 | 5 | 0.88 | 0.84 | 0.91 | 27 |
| 6 | 6 | 0.88 | 0.85 | 0.91 | 43 |
| 6 | 7 | 0.85 | 0.81 | 0.89 | 44 |
| 7 | 1 | 0.85 | 0.82 | 0.88 | 49 |
| 7 | 2 | 0.93 | 0.91 | 0.94 | 58 |
| 7 | 3 | 0.9 | 0.87 | 0.92 | 45 |
| 7 | 4 | 0.9 | 0.87 | 0.92 | 53 |
| 7 | 5 | 0.92 | 0.89 | 0.93 | 49 |
| 7 | 6 | 0.87 | 0.83 | 0.89 | 51 |
| 7 | 7 | 0.8 | 0.75 | 0.84 | 59 |

Note. Songs: 1 = Allegri, 2 = Olafur, 3 = Oblivion, 4 = Explosions, 5 = Vltava, 6 = Hoppipolla, 7 = Brothers; Ratings: 1 = Perceived Sadness, 2 = Perceived Joy, 3 = Perceived Moved/Touched, 4 = Perceived Beauty, 5 = Perceived Connectedness, 6 = Experienced Warmth in the Chest, 7 = Experienced Chills. *Model did not converge.

Table S5. Regression models including the linear and quadratic terms for each rating and song separately.

| Song | Term | B | SE | t | p |
| --- | --- | --- | --- | --- | --- |
| Sadness |  |  |  |  |  |
| 1 | (Intercept) | 1.9872 | 0.064 | 31.00 | 0.000 |
| 1 | Linear | 0.0114 | 0.002 | 5.27 | 0.000 |
| 1 | Quadratic | 0.0000 | 0.000 | -1.83 | 0.069 |
| 2 | (Intercept) | 1.9564 | 0.042 | 46.26 | 0.000 |
| 2 | Linear | 0.0173 | 0.001 | 13.45 | 0.000 |
| 2 | Quadratic | -0.0001 | 0.000 | -8.29 | 0.000 |
| 3 | (Intercept) | 2.0935 | 0.070 | 29.94 | 0.000 |
| 3 | Linear | 0.0335 | 0.002 | 13.92 | 0.000 |
| 3 | Quadratic | -0.0002 | 0.000 | -12.91 | 0.000 |
| 4 | (Intercept) | 1.3748 | 0.028 | 49.96 | 0.000 |
| 4 | Linear | 0.0224 | 0.001 | 29.90 | 0.000 |
| 4 | Quadratic | -0.0001 | 0.000 | -26.80 | 0.000 |
| 5 | (Intercept) | 1.5364 | 0.042 | 36.80 | 0.000 |
| 5 | Linear | 0.0020 | 0.001 | 1.91 | 0.058 |
| 5 | Quadratic | 0.0000 | 0.000 | -2.77 | 0.006 |
| 6 | (Intercept) | 1.3445 | 0.028 | 47.33 | 0.000 |
| 6 | Linear | 0.0040 | 0.001 | 3.53 | 0.001 |
| 6 | Quadratic | 0.0000 | 0.000 | -1.14 | 0.257 |
| 7 | (Intercept) | 1.3747 | 0.070 | 19.62 | 0.000 |
| 7 | Linear | 0.0095 | 0.002 | 4.78 | 0.000 |
| 7 | Quadratic | 0.0000 | 0.000 | -4.38 | 0.000 |
| Joy |  |  |  |  |  |
| 1 | (Intercept) | 1.3670 | 0.043 | 31.68 | 0.000 |
| 1 | Linear | 0.0016 | 0.001 | 1.07 | 0.288 |
| 1 | Quadratic | 0.0000 | 0.000 | 1.56 | 0.120 |
| 2 | (Intercept) | 0.8679 | 0.024 | 35.89 | 0.000 |
| 2 | Linear | 0.0235 | 0.001 | 31.98 | 0.000 |
| 2 | Quadratic | -0.0001 | 0.000 | -25.83 | 0.000 |
| 3 | (Intercept) | 1.2444 | 0.046 | 27.18 | 0.000 |
| 3 | Linear | 0.0086 | 0.002 | 5.42 | 0.000 |
| 3 | Quadratic | 0.0000 | 0.000 | -0.11 | 0.910 |
| 4 | (Intercept) | 1.5049 | 0.052 | 29.15 | 0.000 |
| 4 | Linear | 0.0231 | 0.001 | 16.47 | 0.000 |
| 4 | Quadratic | -0.0001 | 0.000 | -7.95 | 0.000 |
| 5 | (Intercept) | 1.5498 | 0.046 | 33.45 | 0.000 |
| 5 | Linear | 0.0304 | 0.001 | 25.82 | 0.000 |
| 5 | Quadratic | -0.0001 | 0.000 | -19.28 | 0.000 |
| 6 | (Intercept) | 1.2811 | 0.041 | 31.50 | 0.000 |
| 6 | Linear | 0.0835 | 0.002 | 52.00 | 0.000 |
| 6 | Quadratic | -0.0006 | 0.000 | -48.06 | 0.000 |
| 7 | (Intercept) | 2.1823 | 0.086 | 25.28 | 0.000 |
| 7 | Linear | 0.0275 | 0.002 | 11.25 | 0.000 |
| 7 | Quadratic | -0.0001 | 0.000 | -8.62 | 0.000 |
| Beauty |  |  |  |  |  |
| 1 | (Intercept) | 2.0882 | 0.085 | 24.69 | 0.000 |
| 1 | Linear | 0.0079 | 0.003 | 2.79 | 0.006 |
| 1 | Quadratic | 0.0000 | 0.000 | -0.40 | 0.693 |
| 2 | (Intercept) | 1.5157 | 0.032 | 46.96 | 0.000 |
| 2 | Linear | 0.0381 | 0.001 | 38.85 | 0.000 |
| 2 | Quadratic | -0.0002 | 0.000 | -32.00 | 0.000 |
| 3 | (Intercept) | 2.0341 | 0.058 | 34.83 | 0.000 |
| 3 | Linear | 0.0341 | 0.002 | 16.96 | 0.000 |
| 3 | Quadratic | -0.0002 | 0.000 | -11.95 | 0.000 |
| 4 | (Intercept) | 1.8403 | 0.042 | 43.56 | 0.000 |
| 4 | Linear | 0.0274 | 0.001 | 23.90 | 0.000 |
| 4 | Quadratic | -0.0001 | 0.000 | -17.32 | 0.000 |
| 5 | (Intercept) | 1.9811 | 0.059 | 33.40 | 0.000 |
| 5 | Linear | 0.0251 | 0.002 | 16.65 | 0.000 |
| 5 | Quadratic | -0.0001 | 0.000 | -13.65 | 0.000 |
| 6 | (Intercept) | 1.6240 | 0.046 | 35.04 | 0.000 |
| 6 | Linear | 0.0719 | 0.002 | 39.33 | 0.000 |
| 6 | Quadratic | -0.0006 | 0.000 | -37.62 | 0.000 |
| 7 | (Intercept) | 2.1284 | 0.055 | 39.02 | 0.000 |
| 7 | Linear | 0.0262 | 0.002 | 16.98 | 0.000 |
| 7 | Quadratic | -0.0001 | 0.000 | -12.46 | 0.000 |
| Moved/Touched |  |  |  |  |  |
| 1 | (Intercept) | 1.5218 | 0.051 | 29.76 | 0.000 |
| 1 | Linear | 0.0154 | 0.002 | 8.91 | 0.000 |
| 1 | Quadratic | -0.0001 | 0.000 | -4.34 | 0.000 |
| 2 | (Intercept) | 1.0970 | 0.023 | 47.40 | 0.000 |
| 2 | Linear | 0.0371 | 0.001 | 52.70 | 0.000 |
| 2 | Quadratic | -0.0002 | 0.000 | -42.37 | 0.000 |
| 3 | (Intercept) | 1.4653 | 0.041 | 35.88 | 0.000 |
| 3 | Linear | 0.0295 | 0.001 | 20.96 | 0.000 |
| 3 | Quadratic | -0.0002 | 0.000 | -15.29 | 0.000 |
| 4 | (Intercept) | 1.3557 | 0.049 | 27.45 | 0.000 |
| 4 | Linear | 0.0277 | 0.001 | 20.62 | 0.000 |
| 4 | Quadratic | -0.0001 | 0.000 | -12.33 | 0.000 |
| 5 | (Intercept) | 1.5534 | 0.043 | 36.50 | 0.000 |
| 5 | Linear | 0.0257 | 0.001 | 23.79 | 0.000 |
| 5 | Quadratic | -0.0001 | 0.000 | -16.91 | 0.000 |
| 6 | (Intercept) | 0.7976 | 0.033 | 23.88 | 0.000 |
| 6 | Linear | 0.0725 | 0.001 | 55.01 | 0.000 |
| 6 | Quadratic | -0.0006 | 0.000 | -50.55 | 0.000 |
| 7 | (Intercept) | 1.4454 | 0.033 | 43.54 | 0.000 |
| 7 | Linear | 0.0221 | 0.001 | 23.47 | 0.000 |
| 7 | Quadratic | -0.0001 | 0.000 | -13.37 | 0.000 |
| Connectedness |  |  |  |  |  |
| 1 | (Intercept) | 1.2681 | 0.026 | 48.35 | 0.000 |
| 1 | Linear | 0.0137 | 0.001 | 15.45 | 0.000 |
| 1 | Quadratic | 0.0000 | 0.000 | -7.21 | 0.000 |
| 2 | (Intercept) | 1.1580 | 0.029 | 40.50 | 0.000 |
| 2 | Linear | 0.0443 | 0.001 | 51.04 | 0.000 |
| 2 | Quadratic | -0.0002 | 0.000 | -43.49 | 0.000 |
| 3 | (Intercept) | 1.4878 | 0.036 | 41.46 | 0.000 |
| 3 | Linear | 0.0285 | 0.001 | 23.08 | 0.000 |
| 3 | Quadratic | -0.0001 | 0.000 | -16.37 | 0.000 |
| 4 | (Intercept) | 1.5539 | 0.043 | 36.31 | 0.000 |
| 4 | Linear | 0.0249 | 0.001 | 21.44 | 0.000 |
| 4 | Quadratic | -0.0001 | 0.000 | -14.70 | 0.000 |
| 5 | (Intercept) | 1.4796 | 0.037 | 39.93 | 0.000 |
| 5 | Linear | 0.0274 | 0.001 | 29.17 | 0.000 |
| 5 | Quadratic | -0.0001 | 0.000 | -23.22 | 0.000 |
| 6 | (Intercept) | 0.8543 | 0.040 | 21.24 | 0.000 |
| 6 | Linear | 0.0665 | 0.002 | 41.87 | 0.000 |
| 6 | Quadratic | -0.0005 | 0.000 | -35.44 | 0.000 |
| 7 | (Intercept) | 1.5368 | 0.060 | 25.68 | 0.000 |
| 7 | Linear | 0.0270 | 0.002 | 15.93 | 0.000 |
| 7 | Quadratic | -0.0001 | 0.000 | -10.74 | 0.000 |
| Warmth |  |  |  |  |  |
| 1 | (Intercept) | 1.4924 | 0.053 | 28.02 | 0.000 |
| 1 | Linear | 0.0056 | 0.002 | 3.14 | 0.002 |
| 1 | Quadratic | 0.0000 | 0.000 | 1.47 | 0.145 |
| 2 | (Intercept) | 1.2366 | 0.025 | 48.72 | 0.000 |
| 2 | Linear | 0.0276 | 0.001 | 35.84 | 0.000 |
| 2 | Quadratic | -0.0001 | 0.000 | -29.49 | 0.000 |
| 3 | (Intercept) | 1.1628 | 0.031 | 38.09 | 0.000 |
| 3 | Linear | 0.0273 | 0.001 | 26.00 | 0.000 |
| 3 | Quadratic | -0.0001 | 0.000 | -16.13 | 0.000 |
| 4 | (Intercept) | 1.6180 | 0.057 | 28.47 | 0.000 |
| 4 | Linear | 0.0222 | 0.002 | 14.39 | 0.000 |
| 4 | Quadratic | -0.0001 | 0.000 | -8.48 | 0.000 |
| 5 | (Intercept) | 1.3391 | 0.031 | 43.28 | 0.000 |
| 5 | Linear | 0.0187 | 0.001 | 23.80 | 0.000 |
| 5 | Quadratic | -0.0001 | 0.000 | -15.14 | 0.000 |
| 6 | (Intercept) | 1.1903 | 0.024 | 49.33 | 0.000 |
| 6 | Linear | 0.0594 | 0.001 | 62.40 | 0.000 |
| 6 | Quadratic | -0.0004 | 0.000 | -56.17 | 0.000 |
| 7 | (Intercept) | 1.5340 | 0.045 | 34.24 | 0.000 |
| 7 | Linear | 0.0195 | 0.001 | 15.33 | 0.000 |
| 7 | Quadratic | -0.0001 | 0.000 | -9.70 | 0.000 |
| Chills |  |  |  |  |  |
| 1 | (Intercept) | 1.6586 | 0.048 | 34.52 | 0.000 |
| 1 | Linear | 0.0007 | 0.002 | 0.43 | 0.669 |
| 1 | Quadratic | 0.0000 | 0.000 | 2.89 | 0.004 |
| 2 | (Intercept) | 0.8586 | 0.029 | 29.17 | 0.000 |
| 2 | Linear | 0.0291 | 0.001 | 32.54 | 0.000 |
| 2 | Quadratic | -0.0002 | 0.000 | -27.52 | 0.000 |
| 3 | (Intercept) | 1.2044 | 0.027 | 44.82 | 0.000 |
| 3 | Linear | 0.0223 | 0.001 | 24.12 | 0.000 |
| 3 | Quadratic | -0.0001 | 0.000 | -16.04 | 0.000 |
| 4 | (Intercept) | 1.2273 | 0.029 | 42.05 | 0.000 |
| 4 | Linear | 0.0106 | 0.001 | 13.38 | 0.000 |
| 4 | Quadratic | 0.0000 | 0.000 | -3.06 | 0.003 |
| 5 | (Intercept) | 1.1332 | 0.024 | 47.58 | 0.000 |
| 5 | Linear | 0.0159 | 0.001 | 26.29 | 0.000 |
| 5 | Quadratic | -0.0001 | 0.000 | -18.07 | 0.000 |
| 6 | (Intercept) | 0.8914 | 0.025 | 35.49 | 0.000 |
| 6 | Linear | 0.0449 | 0.001 | 45.27 | 0.000 |
| 6 | Quadratic | -0.0003 | 0.000 | -41.37 | 0.000 |
| 7 | (Intercept) | 1.3562 | 0.039 | 34.40 | 0.000 |
| 7 | Linear | 0.0119 | 0.001 | 10.67 | 0.000 |
| 7 | Quadratic | 0.0000 | 0.000 | -5.70 | 0.000 |

Note. Songs: 1 = Allegri, 2 = Olafur, 3 = Oblivion, 4 = Explosions, 5 = Vltava, 6 = Hoppipolla, 7 = Brothers

Table S6. Cross-correlations between feeling moved or touched and the main variables for the sadly moving and joyfully moving excerpts separately. Correlations are presented before detrending and after detrending using the residual, difference and spline (**bold**) methods. Coefficients are calculated using a random effects meta-analysis. Coefficients represent Pearson correlation coefficients.

|  | Sadly Moving |  |  |  | Joyfully Moving |  |  |  | Overall |  |  |  |
| --- | --- | --- | --- | --- | --- | --- | --- | --- | --- | --- | --- | --- |
| Warmth | .94 | .62 | .52 | **.50** | .97 | .71 | .65 | **.61** | .96 | .68 | .60 | **.56** |
| Chills | .93 | .78 | .48 | **.64** | .94 | .67 | .60 | **.60** | .94 | .73 | .55 | **.63** |
| Sense of Connection | .97 | .81 | .55 | **.51** | .93 | .70 | .63 | **.75** | .95 | .78 | .60 | **.66** |
| Perceived Beauty | .94 | .84 | .50 | **.58** | .94 | .73 | .63 | **.60** | .94 | .80 | .60 | **.60** |
| Perceived Sadness | .68 | .54 | .32 | **.49** | -.002 | -.01 | -.05 | **-.33** | .29 | .23 | .11 | **.02** |
| Perceived Happiness | .83 | .54 | .51 | **.49** | .91 | .68 | .66 | **.67** | .88 | .62 | .61 | **.61** |

Table S7. Cross-correlations between feeling moved or touched and the main variables for the sadly moving and joyfully moving excerpts separately. Correlations are presented before detrending and after detrending using the residual, difference and spline (**bold**) methods. Coefficients are calculated using a Fisher-Z transformation. Coefficients represent Pearson correlation coefficients.

|  | Sadly Moving |  |  |  | Joyfully Moving |  |  |  | Overall |  |  |  |
| --- | --- | --- | --- | --- | --- | --- | --- | --- | --- | --- | --- | --- |
| Warmth | .94 | .76 | .52 | **.61** | .95 | .76 | .64 | **.60** | .96 | .76 | .59 | **.62** |
| Chills | .94 | .79 | .47 | **.64** | .96 | .67 | .60 | **.61** | .94 | .73 | .55 | **.60** |
| Sense of Connection | .96 | .81 | .53 | **.50** | .94 | .72 | .62 | **.75** | .95 | .76 | .59 | **.66** |
| Perceived Beauty | .95 | .83 | .49 | **.58** | .94 | .74 | .63 | **.60** | .94 | .78 | .57 | **.59** |
| Perceived Sadness | .74 | .65 | .32 | **.49** | -.001 | .003 | -.06 | **-.34** | .38 | .32 | .11 | **.03** |
| Perceived Happiness | .88 | .58 | .50 | **.53** | .94 | .68 | .65 | **.66** | .92 | .64 | .59 | **.61** |

Table S8. Cross-correlations between feeling moved or touched and the main variables for the sadly moving and joyfully moving excerpts separately at a 1Hz resolution. Correlations are presented after detrending using the cubic spline method. Coefficients are calculated using a random effects meta-analysis. Coefficients represent Pearson correlation coefficients.

|  | Sadly Moving | Joyfully Moving | Overall |
| --- | --- | --- | --- |
| Warmth | .74 [.48, 1] | .69 [.56, .82] | .72 [.59, .84] |
| Chills | .74 [.66, .83] | .68 [.57, .79] | .71 [.64, .78] |
| Sense of Connection | .79 [.66, .93] | .82 [.77, .87] | .82 [.77, .87] |
| Perceived Beauty | .83 [.78, .88] | .75 [.66, .85] | .80 [.76, .85] |
| Perceived Sadness | .83 [.77, .88] | .08 [-.16, .33] | .41 [.08, .73] |
| Perceived Happiness | .59 [.39, .80] | .75 [.69, .81] | .72 [.67, .77] |

Table S9. Cross-correlations between the main variables across all excerpts at a 3s resolution. Correlations are presented after detrending using the cubic spline method. Coefficients are calculated using a random effects meta-analysis. Coefficients represent Pearson correlation coefficients.

|  | Perceived Sadness | Perceived Joy | Perceived Beauty | Sense of Connection | Warmth | Chills |  |
| --- | --- | --- | --- | --- | --- | --- | --- |
| Feeling Moved or Touched | .02 [-.34, .38] | .61 [.48, .75] | .60 [.49. 71] | .66 [.54, .78] | .56 [.32, .80] | .63 [.51, .74] |  |
| Perceived Sadness |  | -.28 [-.64, .09] | .12 [-.21, .45] | -.19 [-.52, .13] | -.07 [-.46, .32] | -.19 [-.56, .18] |  |
| Perceived Joy |  |  | .54 [.37, .71] | .67 [.53, .80] | .68 [.57, .79] | .75 [.71, .80] |  |
| Perceived Beauty |  | . |  | .58 [.46, .70] | .56 [.40, .72] | .51 [.33, .69] |  |
| Sense of Connection |  |  |  |  | .61 [.47, .75] | .61 [.48, .75] |  |
| Warmth |  |  |  |  |  | .65 [.54, .76] |  |


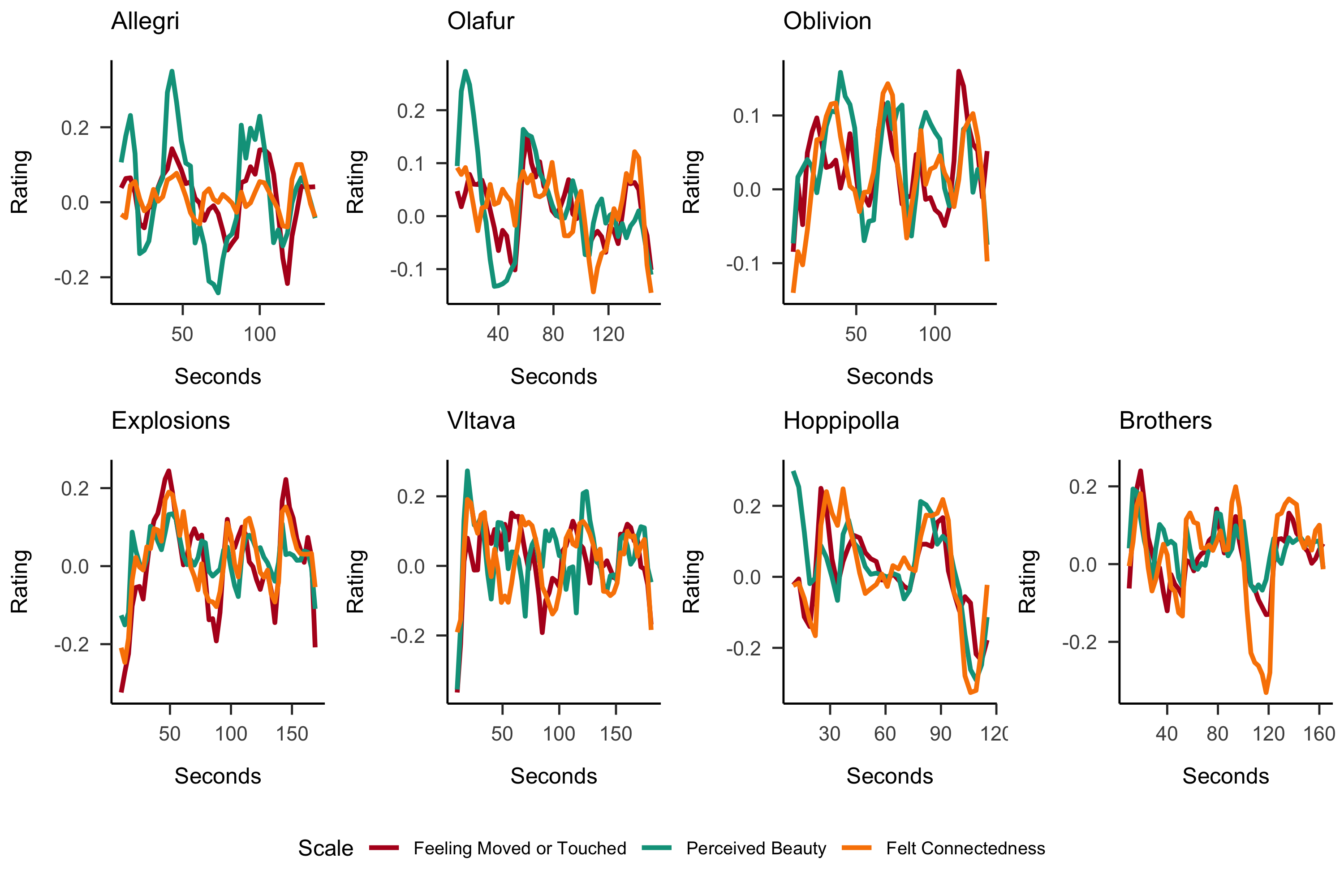


*Figure S6.* The averaged cubic spline detrended continuous ratings of f*eeling moved or touched, perceived beauty*, and *felt connectedness* for the sadly (upper row) and joyfully moving (lower row) music excerpts. Ratings were provided on a 5-point scale (ranging from 1 (not at all) to 5 (extremely)) and aggregated in 3s time bins.


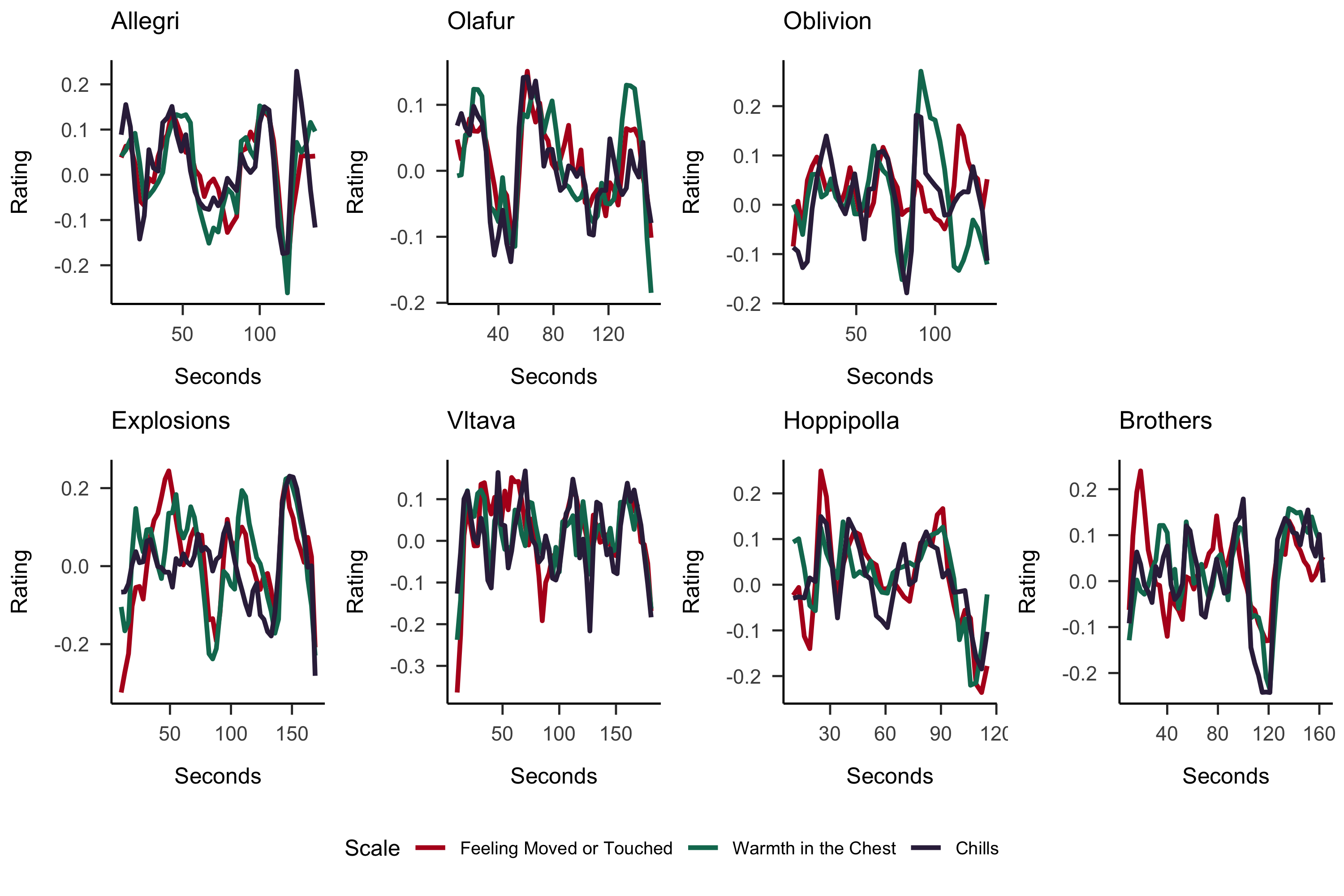


*Figure S7.* The averaged cubic spline detrended continuous ratings of f*eeling moved or touched, warmth in the chest*, and *chills* for the sadly (upper row) and joyfully moving (lower row) music excerpts. Ratings were provided on a 5-point scale (ranging from 1 (not at all) to 5 (extremely)) and aggregated in 3s time bins.


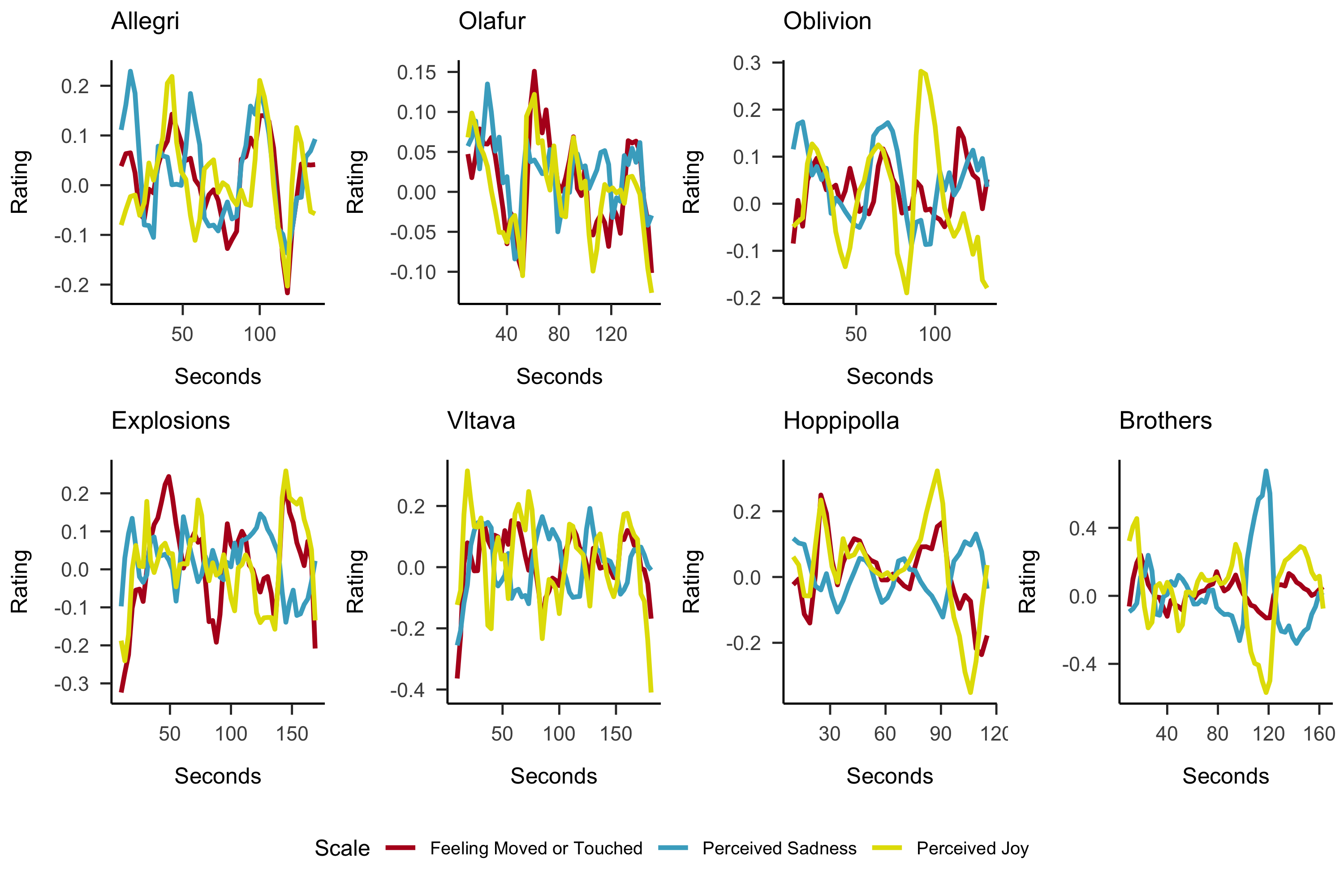


*Figure S8.* The averaged cubic spline detrended continuous ratings of f*eeling moved or touched, perceived sadness*, and *perceived joy* for the sadly (upper row) and joyfully moving (lower row) music excerpts. Ratings were provided on a 5-point scale (ranging from 1 (not at all) to 5 (extremely)) and aggregated in 3s time bins.


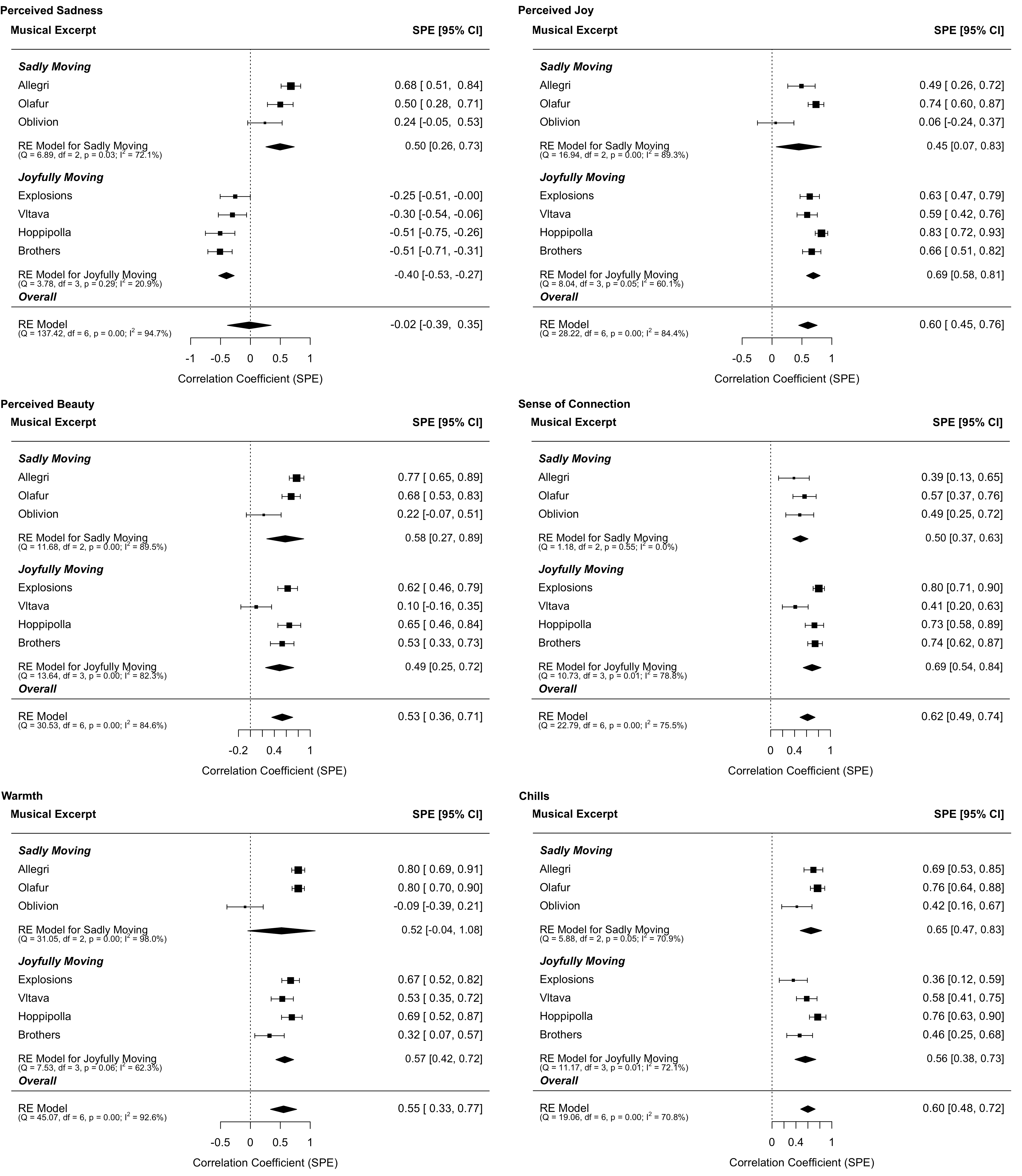


*Figure S9*. Cross-correlation function coefficients (CCF; at lag 0, meaning that both time series are compared at the same time) using Spearman correlation coefficients (SPE) between feeling moved and touched and the six main variables across the musical excerpts and sadly and joyfully moving songs separately employing the cubic spline detrending algorithm. Overall estimates and confidence intervals are constructed employing a random-effects meta-analysis using restricted maximum likelihood estimation. Error bars represent 95% confidence intervals. RE = random effects. As heterogeneity measures we included Cochran’s *Q* and *I2*.

2.3 *Correlations between peak ratings of feeling moved and trait empathy scores.*

We also explore the relations between peak ratings of feeling moved and the two empathy scores. We calculated an estimate across songs by running a random effects meta-analysis for each subscale. Empathic concern correlated significantly with feeling moved or touched *overall* (*r* = .29 [.20, .39])*,* as well as by both *sadly moving* (*r* = .23 [.08, .37]) and *joyfully moving* excerpts (*r* = .34 [.21, .46]). Fantasy showed a smaller non-significant overall effect (*r* = .10 [-.00, .20]), as it did not correlate significantly with feeling moved or touched by *sadly moving* excerpts (*r* = -.02 [-.18, .14]), and only modestly with feeling moved or touched by *joyfully moving* excerpts (*r* = .20 [.06, .33]).

2.4 *Clustering continuous ratings of feeling moved*.

Finally, since the mean ratings of feeling moved or touched revealed significant correlations with trait empathy, we decided to explore individual differences in continuous rating patterns in more detail. It may be that the higher mean values of feeling moved exhibited by the high-empathy participants either reflect higher overall levels of feeling moved, or more pronounced peaks or variations in the continuous ratings. In order to identify potential groups of people with similar rating trajectories, the continuous, non-detrended *moved* or touched ratings were clustered. First, an inter-subject similarity matrix was created using Euclidean distances as a similarity metric. Next, agglomerative clustering was used to reorganize the matrix into nested partitions, thereby creating a hierarchical structure. Ward’s method, which at each step merges the two clusters leading to the least increase in the within-cluster sum of squared distance (between the points in a cluster to its centroid) was used to obtain the structure, as it yields compact spherical clusters when compared to other approaches (Jain & Dubes, 1988). In order to identify the optimal number of clusters, we used the Calinski-Harabasz index (CH), which is similar to the F-ratio in ANOVA in that it seeks to estimate the ratio of between-cluster and within-cluster sum of squares for *k* clusters. Higher values of the CH index denote more stable clustering. We estimated CH index for values of *k* ranging from 2 to 10, and selected the value corresponding to the highest CH index. As a result, the optimal clustering was found to be two for all the pieces except for *Hoppipolla,* which had four clusters. Subsequently, we examined potential differences in the empathic concern (EC) and fantasy (FS) scores of the participants belonging to the clusters. To this end due to the non-normality of the data, we performed Mann-Whitney U tests, a non-parametric test to assess group differences, on the EC and FS scores, which revealed significant differences only for one of the excerpts; *Band of Brothers* (*p* < .05). The participants of Cluster 1 (*Mdn* = 4.42) displayed higher EC scores than Cluster 2 (*Mdn* = 3.85; *U = 133*, *p* = .045). The averaged feeling moved or touched ratings for each of the clusters can be seen in Figure S9. For *Hoppipolla,* due to the presence of four clusters, we use the non-parametric version of ANOVA, that is, the Kruskal-wallis-H test to assess group differences. This revealed statistically significant differences in EC scores, (chi-sq = 12.82, *p* < 0.01). Post-hoc tests revealed that the differences were observed between clusters 2 and 3 (Meanranks = 28.04, 11.5, *p* < .01), and clusters 4 and 3 (Meanranks = 26.77, 11.5, *p* = 0.02) with cluster 3 possessing lower EC scores in both cases. The averaged feeling moved or touched ratings, as well as the median EC scores for these clusters are shown in Figure S9. In the case of both *Band of Brothers* and *Hoppipolla*, the clusters with the highest EC scores were characterized with higher peaks of feeling moved or touched.


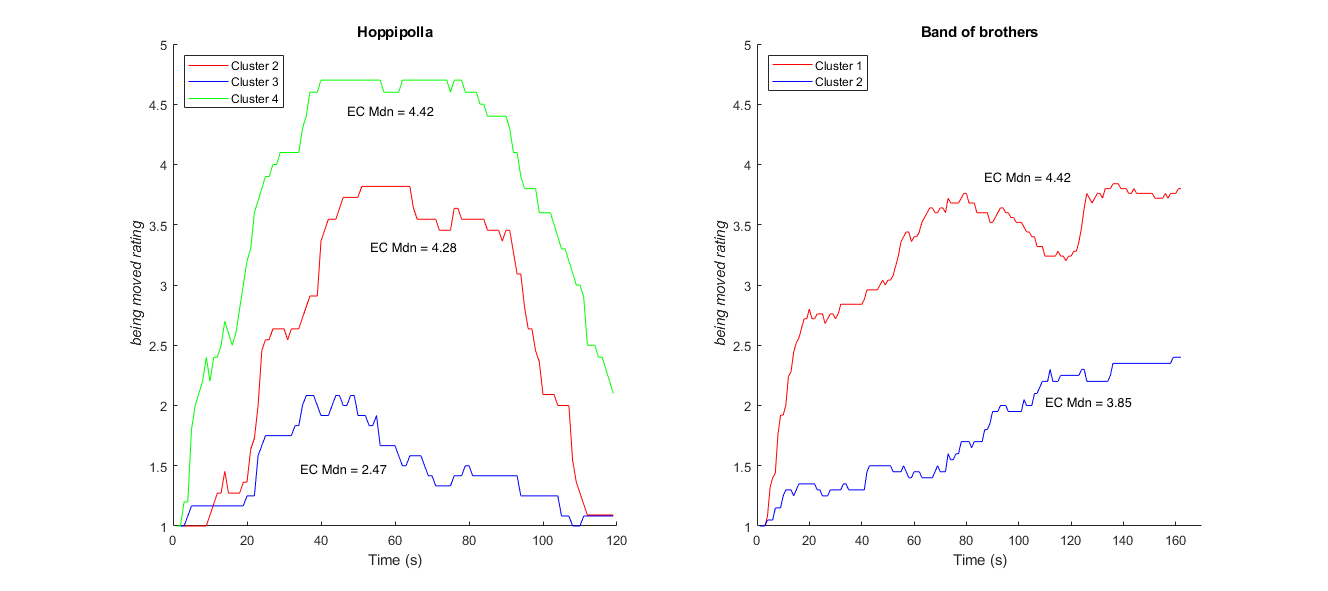


*Figure* S9*.* Cluster-wise averaged continuous ratings of *feeling moved* or *touched* for the clusters that differed in terms of Empathic Concern (EC) scores (median scores displayed in the figure)

***Cross-correlations among all ratings.*** We also explored the cross-correlations among the seven ratings and observed a strong associations among feeling moved or touched, perceived happiness, a sense of connection, reported chills, and warmth, as well as perceived beauty. All effects were above .50 (see Figure 1). Overall, perceived sadness was only to a small degree or negatively associated with the other ratings. Only when considering the *sadly moving* excerpts, this association was stronger, although it never exceeded .49.
