# Supplementary material for: Feeling moved by music: Investigating continuous ratings and acoustic correlates
Source: PLoS One. 2022 Jan 12;17(1):e0261151. doi: 10.1371/journal.pone.0261151 (PMC8754323; doi:10.1371/journal.pone.0261151)
Supplement: Attachment — Submitted filename: Response letter.docx [file pone.0261151.s002.docx]

The comments by the four reviewers have been extremely helpful in guiding our revision process. We agree with many points raised by the reviewers and have restructured our manuscript accordingly.

Specifically, we have revised and streamlined our Introduction to improve its overall clarity and the main goal of our manuscript. In addition, we have restructured the section on different theories of feeling moved, providing a more detailed description of kama muta theory and integrating different theories of feeling moved. Similarly, we have provided more information on our Method and the construction process of the different time series, adding a figure (Figure 1) to visualize this procedure. We have also substantiated our analysis decisions. Finally, we have focused on aligning the Discussion with our findings, interpreting results according to their boundary conditions and highlighting the important limitations of our study.

Below we provide a detailed discussion of the reviewers’ comments and our responses. We provide the reviewers’ comments in *Arial* and our responses in Times New Roman. We have also provided a manuscript version with trached changes.

**Reviewer #1**

*The paper describes one on-line study in which listeners heard 7 pieces of music and provided continuous (or at least, frequently sampled) responses on 7 different scales (sadness, happiness, feeling moved, sense of connection, beauty, warmth, and chills). In general, the ratings across scaled varied similarly over time, however for the songs that were considered “sadly moving” (meaning previously considered to be moving in a sad way), ratings of feeling moved were more correlated with ratings of sadness than ratings of happiness. Conversely, for the songs considered “joyfully moving,” ratings of feeling moved correlated more with ratings of happiness than of sadness. They also showed some correlations between acoustic features for some songs and some rating scales, as well as a correlation between “feeling moved” ratings and subject’s trait empathic concern.*

*The continuous ratings are an interesting way of capturing the real time musical listening experience and it is interesting to see that that distinct rating scales with separate groups of listeners have such similar time courses. In my view the biggest drawback to the paper is an overall lack of clarity in the background or theoretical justification, as well as in the methods. First, the introduction is about twice as long as it should be, and from the start it could be much clearer what questions are being asked and how they fit into what is already known. The literature review is somewhat vague on details, at least for some studies (see specific notes below). I was completely lost on the distinction between “feeling moved” and “being moved” and the “kama muta framework.” What are the differences between these constructs and do they matter for the current study? What is the relationship between “feeling moved” and feeling chills? How is this study novel given that similar approaches have been used to understand acoustic correlates of chills? The entire theoretical background could be shorter, simpler, and clearer.*

We have restructured and revised our introduction to clarify the theoretical background and our research question. We have also shortened the introduction as suggested by the reviewer. We have decided to focus on the state of feeling moved and removed the distinction between ‘being moved’ and ‘feeling moved’. While we think this is an important distinction for theoretical and measurement reasons, it is not as important when reviewing past literature. We have also tried to improve the clarity of our introduction now explicitly referring to the advantages and novel aspects of our contribution. We still introduce kama muta theory as the theoretical backbone of hypothesizing and interpreting our results. Kama muta theory describes an emotion that is often labelled as *moving* or *touching* in English vernacular, but does not necessarily need to be labelled that way as it is mainly based on its appraisal component. Thus, ‘feeling moved’ can be considered the subjective feeling component of the kama muta concept. Specifically we write:

“Kama muta theory defines the emotion of kama muta through the main appraisal theme, intensification of communal sharing. The labels that persons give their emotional experience, such as moved, touched, heartwarming, raptured and others, in English, are but one, though for diagnostic purposes often very important, index for the emotion (Fiske, Schubert, & Seibt, 2017). Conversely, other theories define the emotion through the label “being moved”, encompassing assumed elicitors, subjective feelings, physiological signs and sensations and often also motivational tendencies (see Zickfeld et al. 2019, for a review).” (p. 5)

*My second main concern concerns the clarity of method and how the researchers assessed the validity of the scales. It’s clear that subjects were asked to use 7 different rating scales across 7 pieces, but the paper does not say what they were told about these scales. I’m particularly worried that subjects don’t really have distinct concepts of some of these scales (like beauty vs. feeling moved vs. feeling connected)—how do we know they aren’t just treating multiple scales as being about something like general emotionality or aesthetic intensity or something like that? It helps that sadly moving and joyfully moving pieces showed the distinct patterns that they did, but I’m wondering about the extent to which listeners really understood the rating scales they were using while doing the task. If the same listener had used the same scale on 7 different pieces, then this would at least allow them to compare how that listener tends to use the scale (do they tend to stay high or low on the scale, do they use a wide or narrow range, etc). But the current design doesn’t even allow for within-subject normalization within a given rating scale. It was also unclear to me what happened on a given trial. The manuscript states “participants were presented with 5-point Likert-type scales while listening to the excerpts, and asked to continuously change their ratings while the music progressed.” How did the researchers ensure that they did this? Were they probed every second? Did they have a slider? Please clarify.*

We agree that our original description of our method lacked some important information. We have now restructured the Method section and tried to improve the clarity of our procedure.

In our study, participants were always shown a 5-point Likert-type rating scale and were asked to change their rating when their experience or perception changed. Before starting the continuous self-report tasks, participants received detailed instructions and were able to practice the rating. Afterwards, they were probed whether they understood their task (see <https://osf.io/2dbrg/> for detailed Materials). They were not probed every second during the task but mainly instructed to change their rating when their experience changed. As in 90% of the trials, participants indicated at least one change in the target dimension over the course of the musical excerpt, we are confident that participants performed the task as instructed. We did not exclude participants that did not change their rating for a song x scale combination, as this can genuinely reflect that this particular dimension did not change for them over the course of the musical excerpt.

The validity of our scales was on the one hand tested in our pilot study, which is presented in the Supplementary Material. For example, we replicated previous research that feeling moved or touched correlated positively with perceived beauty (Vuoskoski & Eerola, 2017), but although strong, this correlation did not suggest that these were considered as the same concept. As the reviewer mentions, the observed patterns in our study, especially related to perceived sadness and joy, suggests that our scales were employed in the way we predicted. On the other hand, previous studies investigating feeling moved (e.g., Vuoskoski & Eerola, 2017; Schubert et al., 2018; Zickfeld et al., 2019) have provided evidence that these aspects are associated, but not to the degree that they overlap completely.

We agree with the reviewer that it is a limitation that our data don’t allow for within comparisons. On the other hand, we think it is quite interesting to observe the current cross-correlations for ratings of different participants. We have added a short part to the Limitation section, acknowledging the limitation of our design not allowing for within comparisons:

“Based on our design we were not able to assess intra-individual processes, as participants never rated more than one scale for the same musical piece. All presented comparisons were made on an inter-individual level. While it is intriguing to see the amount of convergence for averaged ratings of different individuals for the same musical excerpt, this approach might have overestimated correlations among the different ratings, as within error variance remains undetected.” (p. 37)

*p. 3, first paragraph: I do not understand the distinction that is being made between “culturally and socially constructed” emotional interpretations of music and an “embodied, empathic basis” for musical emotion. Can’t it be both?*

We have restructured the introduction and removed the paragraph referred to by the reviewer.

*p. 4: the claim is that music can express “social attitudes” but the study described used forced choice responses. It isn’t clear that subjects would spontaneously use terms such as “disdainful” to describe a musical performance, and it’s quite possible they are responding to more general emotional categories in this task.*

We have restructured the introduction and also the paragraph referred to by the reviewer. Our main focus was on the finding that music can express affiliation and not so much related to the specific attitudes such as “disdainful”. Otherwise, we agree with the reviewer that the force-choiced design might not reflect spontaneous evaluations of music performance.

*p. 4: I think the authors meant to cite a different paper than Mehr & Spelke (2018)-- perhaps the Mehr (2018) Current Biology paper?*

We have restructured the introduction and removed the paragraph referred to by the reviewer.

*p. 9-10: What is meant by “the opposite direction” re: chills? Some studies find that chills are frequent and others find that they are infrequent? The summary on chills is very unclear.*

Our main intention with this paragraph was to convey that for some types of methods chills seem to occur frequently in response to music, but frequency is reduced in some experimental studies. We have removed this part as we agree that it was more confusing than informative. In general, we have restructured the paragraph on chills and think that it is more to the point now.

*p. 15: why were American subjects recruited for this study?*

We only requested US American participants, as emotional responses to music might be influenced by different cultural backgrounds. As there are many Indian participants in the workers’ pool on Amazon MTurk, we applied this criterion. We now explicitly write:

“We excluded non-US participants to ensure that the participants had a shared, consistent understanding of the emotional labels and concepts used in the rating tasks, and to control for possible effects of cultural background on emotional experiences in response to music (cf. Laukka et al., 2013).“ (p. 16)

*p. 16: The osf link just goes to a spreadsheet and some R code. Are the stimuli or a stimulus available somewhere?*

We apologize for the confusion. The stimuli are now available on our project page: https://osf.io/wjpqg/.

*p. 20: the cross-correlations for ratings are over means across all subjects who used the rating scale, correct? No individual subject time series are being used?*

Yes, this is correct. As mentioned in the data preparation section:

“Second, we averaged the time series for each scale-excerpt combination across participants, resulting in seven averaged time series ratings for each of the seven excerpts (Figure 1, 2). ” (p. 19)

As participants only completed one scale for the same musical excerpt, comparison of individual subject time series was not possible given our design.

*p. 24: it would be helpful to have a little more information about the acoustic measures that were used. More is said in the discussion but it would be good to also have it in the results section (like, what does zero-crossing rate mean).*

We have added a table (Table 1) providing a short overview of the acoustic measures and their descriptions.

**Reviewer #2**

*This is a very interesting paper by very esteemed scholars. It proposes a very strong methodology for rating emotional reactions “while” listening, and not “after” listening. Also the search for underlying psychological mechanisms is interesting, especially the time specificity of the reactions. The strength of the paper, however, is also its mean weakness. The methodology is very strong but is too technical to be understood by common readers. A major effort should be made therefore to make the paper more readable and understandable by providing more intuitive descriptions of the technicalities, without giving in on depth of elaboration. As I consider this paper to be a very valuable contribution, I did a very demanding review in order to be as critical-constructive as possible. It means also that I would like to see this paper to be published on condition that my remarks and comments are addressed appropriately.*

*Note: I have commented on the paper in the order of reading, to show what readers may experience as problems why reading. Many of my remarks are addressed and even solved at later passages in the text, but for the noninforemed listerern this comes too late. In that case, it could make sense to move these passages to earlier places in the text to make reading more easy. I therefore decided not de delete these comments. I did also a first reading of the paper without reading the supplementary materials to have an idea of the readability of the paper. This was a hard but very instructive exercise.*

*General remarks*

*• Language use is OK.*

*• Authors show sufficient knowledge of the field.*

*• The reference list is quite exhaustive though some reference to real-time listening and musical emotions could be added.*

Based on your and the other reviewers’ comments, we have added additional references on continuous ratings of musical stimuli (e.g., E. Schubert, 2010) and research on musical emotions (e.g., Zentner et al, 2008; Coutinho & Scherer, 2017).

*• The methodology, though sound, is not well described. It could be explained more clearly by providing a more schematic overview of the major research questions. This holds in particular for the way how the continuous rating is assessed. Even after reading the whole paper this is not clear as a take-home message.*

We agree that our original description of our method lacked some important information. We have now restructured the Method section and tried to improve the clarity of our procedure. Specifically, we now describe the overall procedure and method first and later introduce the different materials and measures.

The description of our procedure now reads:

“The present experiment followed a 7 (within: musical excerpt) x 7 (within: rating scale) mixed design. Each of the seven excerpts was randomly coupled with one of the seven rating scales for each participant. More specifically, for the first excerpt and rating, participants were randomly presented with one out of 7x7 possible combinations. For the second excerpt, participants were randomly presented with one out of 6x6 possible combinations and so on. The type of combination and order of all excerpts and ratings was randomized for each participant individually. Importantly, each excerpt and rating scale was only presented or used once by each participant.

The general continuous self-report paradigm was based on T. W. Schubert et al. (2018). The study was created in Qualtrics. After providing informed consent, participants were presented with detailed instructions about their task and were familiarized with the continuous rating scales. They were then presented with the seven different combinations of musical excerpt and rating scale in individual random order. Before each excerpt, they were informed what aspect they should rate and reminded about how to do that. Excerpts played automatically and participants were instructed to rate their experiences or perceptions continuously, changing their rating when their experience or perception changed. For that purpose, participants were shown a Likert-type rating scale while listening to the excerpt. Each scale included five scale points ranging from ‘'Not at all (1)', 'A little (2)', 'Moderately (3)', 'Very (4)', to 'Extremely (5)' (with numbers in brackets referring to the scale point or the specific number key; see below for a specific example). Participants were instructed to update their response by either clicking on an option with their mouse, using the arrow keys to decrease or increase their rating, or using the number keys on their keyboard to indicate a response between 1 and 5. The lowest scale point was selected as the default when participants started a new excerpt (an example of the rating screen is provided in the Supplementary Material).

We retained the exact time at which participants changed their rating and matched it to the time code of the musical excerpts using a JavaScript code. Musical excerpts were hosted on YouTube and embedded in the survey by hiding player controls and visuals.“ (p. 16-17)

*• The readability of the paper is rather low, given the many technical terms without clear description. At times the reader has the impression that this is a method-driven paper rather than a paper about interesting and clear contents. There is, as such, a kind of imbalance between empirical facts and data (measurements) and clear intuitions and take-home messages. There is a lot of technical stuff, which is not sufficiently explained, even for readers with a lot of knowledge of the field. The authors should keep in mind that to understand the paper readers should be a kind of combination of acousticians, mathematicians, engineers, statisticians, and musicians. Given the interdisciplinary team of authors, this is no problem for the authors, but it is very hard to find readers who combine these competences. Therefore, I emphasize repeatedly the need of clear and intuitive descriptions of the intuitions behind the measurements so as to reach a broader readership.*

We have reduced the technicality of our Method and Result section and now provide examples of each technical term introduced.

*• The clarity of the paper could be better. At times it is difficult to understand the methodology with the needed description being provided “after” the facts. Some restructuring is necessary to help the reader to understand the design and the assessment.*

As mentioned earlier, we have restructured the paper, especially the Method section and think that the description of our study is now clearer.

*• Some very technical procedures are not explained. Provide at least some intuitive descriptions (e.g. the Interpersonal Reactivity Index, detrended correspondence analysis, cubic spline detrenching, Ward’s minimum variance method , Calinski-Harabasz index, Mann-Whitney U test, Kruskal-Wallis-H test, etc.) as not all readers have the needed statistical background to understand the methods used. It is perhaps a suggestion to insert a box with some technical terms and their explanation to make the paper more readable.*

We have reduced the technicality of our Method and Result section and now provide examples of each technical term introduced (for example by adding Table 1). Additionally, we always do refer to literature providing a detailed description of these technical terms.

*• The time-series must be explained more clearly, given their importance throughout the paper.*

We have revised the description of our data preparation strategy and have included a Figure (Figure 1) in order to visualize the process of constructing the final time series. We think that this has improved clarity regarding the main process. In addition, we now describe this process in detail in the ‘Data preparation’ section on pages 19-22.

*• The figures are very interesting and insightful. In order to be more easily interpretable, however, they must be explained better, either in the main text or in the figure captions. This holds especially for the abbreviations and the units used. A figure, together with its figure caption, must be understandable at least to some extent without reading the main text.*

We have revised the Figures accordingly and added more information about abbreviations in the figure captions. They are now presented as Figures 3-5.

*• Some concepts are introduced without clear description (e.g. self scale, zero-crossing rate, entropy): some intuitive descriptions should be provided to guide those readers who are not familiar with the field.*

We now provide short descriptions of the various measures. For example, when referring to the inclusion-of-the-other-in-the-self scale we write:

“[...] or with the inclusion-of-the-other-in-the-self scale measuring closeness by showing two circles increasing in overlap” (p. 13)

*The concepts of kama muta needs a clear definition.*

We tried to introduce the kama muta concept in more detail in the introduction (p. 4-5). However, a detailed description and comparison to similar concepts of feeling moved is beyond the scope of this paper and can be found in papers specifically devoted to this matter (e.g., Fiske et al., 2017; Zickfeld et al., 2019; Cullhed, 2020). In the Discussion section we devote more space to discuss the results in light of the different theories.

*• The major question how the continuous rating was assessed is not explained sufficiently clear. It seems hard to imagine how listeners can fill in a rating for a scale every second or something like this. Even after reading the whole paper, this procedure is not clear. It should be explained much more clearly, given the importance of the procedure for the whole paper and its interest for the broader readership which I consider to be very great.*

As mentioned earlier, we have restructured our Method section and now present the continuous rating task in more detail. Participants were not filling in the scale every second, but asked to change their ratings when their perceptions or experiences changed. We then retained their measurements at 1Hz (i.e. one rating per second).

*• There is an inflation of statistical procedures and techniques which make reading very hard and which do not always add much to the main findings. There is a risk of method-driven analysis with the danger that the reader does no longer see the forest through the trees.*

As mentioned earlier, we now provide more detailed examples for technical procedures. On the other hand, the main goal of our article is to make inferences about responses to moving music and for that reason it is necessary to employ statistical procedures. The paper is divided into confirmatory and exploratory analyses. The confirmatory part, the comparison of time-series, was registered so these analyses are not method-driven. The exploratory analyses are jointly driven by the goal to find characteristic musical features predicting feeling moved responses and by the available methods for extracting musical features. To reduce the amount of exploratory analyses we have moved some parts such as the cluster analysis of the empathy ratings to the Supplementary Material.

*Note: I have listed my comments in order of appearance while reading the paper. Some questions or remarks are addressed appropriately, but they come too late, which makes it difficult for the reader to understand the methodology. Some restructuring and moving of some sections to earlier places in the paper seems necessary therefore. Now, as a reader, we have the impression to be informed sometimes after the facts.*

*Detailed comments*

*• line 43: it is not common to insert references in an abstract; the abstract is also rather long*

We have removed the reference from the abstract and shortened the abstract while trying to retain the most important information.

*• line 69: additional references to musical emotion could be added here (see e.g. Juslin & Laukka, 2004; Reybrouck & Eerola, 2017)*

We have restructured the introduction and removed the specific sentence referred to by the reviewer.

*• line 132: please explain clearly which are the three aspects of the aesthetic trinity. This is not clear.*

We have explicitly mentioned the two additional aspects of the *aesthetic trinity*: chills or thrills and aesthetic awe:

“Konečni (2005, 2011) treats being moved as part of the *aesthetic trinity,* next to *thrills* or *chills* and states of *aesthetic awe.*” (p. 5)

*• line 138: is it possible to describe the concept of “kama muta” still more in detail, given its importance for this paper and given that the concept is not commonly known*

We have tried to devote more space to the introduction of the kama muta concept, while at the same time trying not to increase the length of the Introduction even more. As mentioned earlier, a detailed introduction and comparison with other theories is beyond the scope of the present paper. We now emphasize that other theories of feeling moved make comparable predictions, something we also take up in the Discussion.

*• line 144: the four relationship types are summed up but are not explained. Is it possible to give a very short intuitive description of each of them?*

We feel that for the purpose of the current manuscript it is not essential to introduce the remaining relational models in detail. We highlight that they are “expressed by giving according to hierarchy, equality, and proportionality” (p. 4), but think that in order to avoid blowing up the introduction even more a more detailed introduction is not necessary. We now explicitly refer to Fiske (2004) for an in depth treatise of the remaining models.

*• line 147: what is commensalism (an association between two organisms in which one benefits and the other derives neither benefit nor harm)? what is consubstantial assimilation? Introducing such terms without defining them seems somewhat pedantic and hampers the readability of the paper.*

With commensalism we refer to the practice of eating together or sharing food (see Fiske, 2004). We have now replaced this term with ‘food sharing’ to clearly illustrate what we mean.

*• line 165: do the correlation coefficients refer to either experienced kama muta and trait empathic concern? In that case insert them after these words to avoid confusion. What do the number between square brackets stand for? Is it the interval between the lowest and highest value? the confidence interval? Please indicate this to help the reader. Even for readers with considerable scientific training, the statistics and their abbreviations are sometimes elusive.*

We have restructured the introduction and removed the reference to the specific statistics as referred to by the reviewer. Originally, the number in brackets referred to the 95% confidence interval.

*• line 183: feeling moved. Why using italics here? Is there something to be stressed?*

The referred formatting was unintended and we have removed it.

• *line 183: style of referencing: more than three authors: et al.?*

We have changed the reference accordingly.

*• line 192: why semicolon after musical pieces?*

This represents an error that has been corrected accordingly.

*• lines 198-199: this sentence is not clear. Is there a hyphen that is lacking?*

We have restructured this sentence and hope that it is now more intuitive.

*• line 271: the concept of computing time series seems to be quite challenging. However, after having read the whole paper, it is not yet clear how this is actually done. This should be explained very clearly at the first appearance of the term.*

*• line 283: same remark. How is the continuous rating done in practice? Not clear. What must the listeners do? Have they to fill is something every second on a list? Must they check boxes or Lickert scale or write down words? This must be explained very clearly from the beginning in order not to affect the readability.*

Related to both comments above, we think that it is most meaningful to introduce the actual procedures of the continuous self-report paradigm in the Methods section. We have also restructured parts of the Introduction so that the detailed discussion of T. W. Schubert et al. (2018) was removed. We mainly mention that we are using a continuous self-report paradigm in the introduction and briefly highlight that responses are made *while* listening to music and not *after*. We also indicate briefly that responses were made on a Likert-type scale and participants were instructed to change their ratings when their experiences/perceptions changed. We think that we do not have space to introduce the task in detail twice.

*• line 289: it seems that this paragraph relates to the major research questions of the paper. Perhaps this should be stated more explicitly to make the methodology more transparent.*

Line 289 as referred to by the reviewer revolved around advantages of continuous self-report paradigms in comparison to common post measurements. This aspect is a main feature of our design, but does not relate to the major research question. Our major research question is: How is feeling moved elicited by music? More specifically, in the confirmatory part we ask: What impressions about the musical piece and what sensations are most closely related to feeling moved? And in the exploratory part: What specific acoustical or musical features elicit feeling moved?

*• line 294: as readers we are curious about how this continuous assessment did happen. Please explain.*

As mentioned earlier, we have briefly introduced the main background of the method. However, as presented above we think that the exact method should be introduced in the Methods section.

*line 296: what is the motivation behind the selection for these items?*

The items were selected because they represent the main emotional components of kama muta. Chills and warmth refer to the most common physiological or bodily responses (we also include tears, but not as a continuous measurement). Sense of connectedness refers to the appraisal. Moved or touched refer to the feeling component. In addition, we included beauty because previous research has found a link between feeling moved and perceived beauty for music. Perceived happiness and sadness are employed to assess another aspect of the feeling component. We now write:

“Seven variables or ratings were assessed continuously for all excerpts to target the feeling, physiological, and appraisal components of the state of the emotion. To assess the feeling component, we included an item of *feeling moved or touched* as used in a previous study (T. W. Schubert et al., 2018). For the physiological component, we included items on *chills* and feelings of *warmth (in the chest)* (based on T. W. Schubert et al., 2018). The quality of warmth in the center of the chest has been strongly associated with feeling moved (e.g., T. W. Schubert et al., 2018; Zickfeld, Schubert, Seibt, Blomster, et al., 2019), while there has been only limited research exploring the reaction in response to musical stimuli (e.g., Bannister, 2019). At the moment it is not clear whether these feelings of warmth in the chest are associated with an actual objective temperature increase, or whether participants embody the metaphor of communality and feel social warmth (see Inagaki, Irwin, Moieni, Jevtic, & Eisenberger, 2016; Nelson & Panksepp, 1998; Zickfeld, Schubert, Seibt, & Fiske, 2019). Note that we did not assess the common response of tears or moist eyes continuously (this was rated only after the music excerpt had ended), since our pilot study indicated a rather low occurrence of self-reported tears or moist eyes in response to our stimuli. For the appraisal component, we wanted to assess the affiliative signals or communal sharing aspects conveyed by the music. While previous studies have measured this aspect using either items with regard to how close someone feels to another target (Zickfeld, Schubert, Seibt, Blomster, et al., 2019), how close the protagonists are to each other, or with the inclusion-of-the-other-in-the-self scale measuring closeness by showing two circles increasing in overlap (Aron, Aron, & Smollan, 1992; T. W. Schubert et al., 2018), we felt that it might seem artificial to probe how close someone feels to a certain musical piece while it unfolds. Based on the findings of a pilot study, we decided to assess the extent to which the participants felt a sense of connection when listening to music, relating to findings that individuals are able to detect affiliative intentions in musical stimuli (Aucouturier & Canonne, 2017). Given that moving music is perceived as more beautiful than non-moving music (Vuoskoski & Eerola, 2017; Hanser et al., 2021), we also included an item on perceived beauty in order to test whether continuous ratings of beauty are similar to ratings of feeling moved. Finally, in line with previous studies, we also assessed how *sad* and *joyful* participants perceived the music. Specifically, we targeted the emotions perceived in the music and not felt emotions for these two items, although it should be noted that the two processes are strongly related (cf. Vuoskoski & Eerola, 2017). This choice was made due to the possibility that the continuous ratings of experienced joy could be confounded by enjoyment of the music as a result of being moved by the music. We were, however, more interested in the elicitors of feeling moved or touched.” (p. 12-13)

*• line 301: here a reference could be made to the mechanisms of thermoregulatory behavior as studied e.g. by Panksepp and others. This is only a suggestion.*

We have added a reference to Nelson & Panksepp to this paragraph as suggested by the reviewer:

“At the moment it is not clear whether these feelings of warmth in the chest are associated with an actual objective temperature increase, or whether participants embody the metaphor of communality and feel social warmth (see Inagaki, Irwin, Moieni, Jevtic, & Eisenberger, 2016; Nelson & Panksepp, 1998; Zickfeld, Schubert, Seibt, & Fiske, 2019).” (p. 12)

*• line 305: is there a motivation behind the choice for “emotions perceived” rather than “felt emotions”?*

We have added a short justification why we focused on perceived emotions in the case of happiness and sadness. Our main goal was to reduce the conflation of participants' ratings of their own happiness with the degree to which they enjoyed the music. As mentioned, there is a high overlap between ratings of felt and perceived emotions in research on music. Nevertheless, we have added a short paragraph in the Limitation section in order to acknowledge the shortcomings of our decision to assess perceived rather than felt emotions:

“Specifically, we targeted the emotions perceived in the music and not felt emotions for these two items, although it should be noted that the two processes are strongly related (cf. Vuoskoski & Eerola, 2017). This choice was made due to the possibility that the continuous ratings of experienced joy could be confounded by enjoyment of the music as a result of being moved by the music. We were, however, more interested in the elicitors of feeling moved or touched.” (p. 13)

*• line 313: please provide a short description of what is meant with the self-scale*

We have added a short explanation that the IOS scale measures closeness by showing two circles increasing in overlap:

“or with the inclusion-of-the-other-in-the-self scale measuring closeness by showing two circles increasing in overlap (Aron, Aron, & Smollan, 1992; T. W. Schubert et al., 2018).” (p. 13)

*• line 315: does it make sense to refer here to a conception of music in terms of agency?*

We have revised this sentence, trying to explain that participants rated their felt sense of connectedness. This could refer to a sense of connection conveyed by music, but does not necessarily need to:

“Based on the findings of a pilot study, we decided to assess the extent to which the participants felt a sense of connection when listening to music, relating to findings that individuals are able to detect affiliative intentions in musical stimuli (Aucouturier & Canonne, 2017). ” (p. 13)

*• line 329: this paragraph addresses the previous comment regarding line 289. Perhaps the layout could be changed into a bulleted/numbered list to make the claims more visible as research questions or predictions*

We agree with the reviewer and have changed the outline into a bullet point list.

*• line 332: the second prediction is not clear. What is meant precisely?*

With the second prediction we argue that the dynamic unfolding of the feeling component of feeling moved should correlate positively with the dynamic unfolding of perceptions of beauty. Differently put, felt moved or touched is expected to show the same time course as perceived beauty.

*• line 343: the difference between empathic concern/fantasy and being moved is not totally clear. Please explain a little more in detail. Does it make sense to refer also to “theory of mind” in this context?*

*Fantasy* refers to the tendency to take the perspective and feelings of fictitious characters. *Empathic concern* refers to the tendency to show sympathy and compassion for others in need. *Feeling moved* refers to the subjective feeling component of an emotional state (labelled as *kama muta* by some researchers or *being moved* by others). The first aspects refer to trait concepts, the latter one to a state. The fantasy scale could reflect parts of *theory of mind* (Davis, 1980). However, this is typically more attributed to the *perspective taking* scale of the Interpersonal Reactivity Index (IRI, Davis, 1980), which was not included in the present study. *Empathic concern* refers to something that has been called *affective empathy*. Thus, we do not think that a discussion of *theory of mind* is needed in the present context.

*• line 365: explain what is meant here with “cell”? Which cell? What is grouped together in these cells? A short intuitive description is very important to guide the listener. Where do the 7 ratings come from? This is not yet explained and should be done also. Referring to the supplementary material is not enough here? The main text should be understandable also on its own.*

As we employ a factorial design (7x7), the combination of independent variables is typically referred to as a *cell*. We have now added a short description and explanation of the term cell. We already introduce the seven ratings in the Introduction (p. 12-13), so we think this should be understandable (see also reply to previous comment).

*• line 370: approval rating: approval of what? Please be clear.*

We have added a short part to clarify that this refers to the approval ratings that *workers* on Amazon MTurk get for finished tasks.

*• line 376: it seems as if the reader should have knowledge of the rating here, but up to here, there has not been any description of these ratings, which makes reading and understanding very difficult.*

As mentioned above, the different ratings are introduced in the Introduction section (p. 12-13; see earlier reply).

*• line 381: same remark as above. Provide at least a short description of the music excerpts. Substitute also “music excerpts” for “excerpts”.*

We have changed “excerpts” for “musical excerpts” as suggested by the reviewer.

*• line 389: this is the answer to the previous comment; perhaps it makes sense to add “see below” at the first referenced to the music excerpt.*

We agree with the reviewer that it is important to clarify different aspects as they are introduced. However, in this case a more detailed description of the musical excerpts is provided three sentences later. Likewise, we think that cross-reference can also distort reading flow that is why we have kept the paragraph as it was.

*• line 398: here at first there is a description of the scales, but this is also not yet sufficiently clear. What are the units of each scale? How are they measured in a continuous way or using a Lickert scale? An example here could be very helpful.*

As mentioned earlier, the scales are already introduced in the Introduction (p. 12-13; see earlier reply). Based on the reviewer’s feedback we have also provided more information on the continuous procedure and the type of scale that is used. An example of the rating task is also provided in the Supplementary Material (Figure S4).

*• line 407: here is the answer to the previous remark. This comes too late, or at least there should have been made a reference to this at an earlier place in the text.*

Based on the reviewer’s feedback, the Method section has been restructured so that information about the continuous rating procedure is presented first.

*line 420: major question: how is this continuous rating done? Please explain the procedure. Is there a time grid? This is not clear?*

As mentioned in an earlier reply, we have now clarified the continuous rating task.

*• line 429: even though I have some considerable knowledge of statistics, it is not clear what the symbols between the brackets stand for: does ‹ mean lesser than or something like this? Please explain a little in intuitive terms what the Interpersonal Reactivity Index is and how we must interpret number given between brackets.*

This was an error and we thank the reviewer for pointing it out. The symbol in brackets should have referred to Chronbach’s ɑ. We have changed this accordingly. In addition, we have provided a sentence on the focus of the Interpersonal Reactivity Index:

“Upon completing the ratings for all seven excerpts, participants were asked to complete the empathic concern (Chronbach’s ɑ = .91) and fantasy (ɑ = .85) subscales of the Interpersonal Reactivity Index (IRI; Davis, 1980), a questionnaire designed to assess interindividual differences in empathic responding. ” (p. 19)

*• line: it is not clear how the time series was created. Please explain the timestamped rating procedure. Does this mean that the participant that the subject had to rate on a scale every second? This seems rather hard to do in real time.*

We have revised the description of how the time series was created. To visualize our process we have included a Figure (Figure 1). Participants did not rate the scale every second, but mainly we transformed participant ratings to reflect a resolution of 1Hz (or one rating per second). We have included a short description of that process:

“For example, if a participant started with a rating of 1 on a specific scale and changed their rating to a 3 at 30s the time series would show a rating of 1 from 0 to 29s and a rating of 3 from 30s until the second the rating was changed again (or until the end of the musical excerpt if the rating did not change). ” (p. 19)

*• line 447: what is meant with “we decreased the resolution of the time scale”. It seems that this is all very important stuff, but not enough efforts are done to explain the intuitions behind for the non-informed reader. Reference to the supplementary material can help of course, but the paper must be understandable also on its own.*

As mentioned earlier, we have included a more detailed description of the time series creation process (Figure 1). Decreasing the resolution of the time scale referred to binning ratings according to a certain value, in our case 3s. As ratings were originally recorded at a 1s resolution, ratings performed for 1-3s, 4-6s, 7-9s and so on were averaged so that we retained only one rating per time bin.

*• line 449: increases/decreases over time: what kind of increases/decreases? Please specify.*

This refers to increases or decreases in the specific ratings. We have added this information to the text.

*• line 455: detrended correspondence analysis is a rather technical field, not commonly known to common readers of the journal. Please provide at least some short intuitive description of the technique and how to interpret the results. Explain in intuitive terms what detrending means [the word deterendin can even not be found in an English dictionary], what it is used for and how it offers an additional value for the interpretation of the data. A graphic example could be helpful here, also for explaining the construction of the time series. It seems that such an important aspect of the paper does not come into its own sufficiency by presenting it in a manner that is much too technical and not really understandable. Musicians, and even music psychologist, are not statisticians, mathematicians, or engineers, even if the latter may contribute largely to the computational parts of many papers.*

We could not find the term ‘detrended correspondence analysis’ in the manuscript as suggested by the reviewer. Further, in line with the suggestion, we have now included a figure (Figure 1) to visualize the process of detrending as suggested by the reviewer. We have also included a short description of why detrending is important:

“Fourth, we detrended the time series. Most time series are non-stationary, meaning that they show linear or higher-order increases or decreases. Comparing two non-stationary time series would inflate correlations, as they tend to cross-correlate simply due to the fact that they have trends in common. Detrending, removing the trend in time series, is one possible way to achieve the stationarity of time series data (Wu et al., 2007; Shumway & Stoffer, 2011). Inspecting our time series, all averaged curves showed increases in ratings over time and some also decreases in ratings towards the end. More specifically, they indicated positive linear and negative quadratic trends. We detrended all time series using a cubic spline interpolation (Wu et al. 2007; McKeown & Sneddon, 2013).” (p. 20)

*• line 458: same remark. What is “cubic spline detrending”? I am afraid that readers will stop reading here, because of the technicality of the terms. See my suggestions above to insert a box with technical terms and their description.*

We have included a short description of the cubic spline procedure:

“We detrended all time series using a cubic spline interpolation (Wu et al. 2007; McKeown & Sneddon, 2013). Splines can be considered as an extension of polynomial regression that divides the time series into a number of k intervals, delimited by *knots*. Employing a cubic spline, a regression with three parameters (linear, quadratic, and cubic) is fit at each knot. We used five knots and therefore six intervals for all time series (Lusa & Ahlin, 2020; Figure 1, 4). “ (p. 20)

Unfortunately, the scope of our paper is beyond a detailed discussion of detrending techniques and we refer the interested reader to more detailed methodological papers.

*• line 461: please specify what was the residual method*

The residual method is now explained in more detail. We have restructured this part of the paper and now provide a short description of the procedure of the residual detrending method:

“For the residual method, we removed the linear and quadratic trends from all curves by regressing them separately in a multiple regression on an index of time in seconds and its square, and saved the unstandardized residuals (Shumway & Stoffer, 2011; Table S5)” (p. 20)

*line 462: what is meant with “pre-registration” here? This seems a strange wording in this context, which is rather confusing.*

“Pre-registration” here refers to the registration of analysis procedures and decisions before performing the analyses (e.g., Nosek et al., 2018). We have mentioned the fact that our hypotheses and the confirmatory analyses were registered prior to conducting the study in the Introduction. We have revised this sentence in order to make clear what we are referring to.

*• line 470: please specify which 11 acoustic features were chosen.*

We have added a table (Table 1) to provide an overview and description of the 11 acoustical features.

*• line 472: two categories: which ones? Please specify them shortly before elaborating on them.*

*• line 475: here the previous remark regarding line 470 is solved. Perhaps it can help the listener by changing line 472 into “The features are listed below and can typically…”*

As mentioned earlier, we think that too much cross-referencing can disrupt the reading flow, especially when the specific aspects are explained within the next two sentences.

*• line 477: same remark as above; the acoustic features are also rather technical. Yet they are quite important. Perhaps also here a box can be introduced to provide a short description of these terms rather than referring to the supplementary material. This is only a suggestion. In the other case, the terms can be explained more in depth in the supplementary material.*

We have added a table (Table 1) to provide a description of the 11 acoustical features as suggested by the reviewer.

*• line 484: here again, the previous remark has been addressed appropriately. Perhaps it makes sense to move this reference to the MITtoolbox already to line 471.*

We think that the addition of the table describing the different musical excerpts helps to get an overview of the different features. Therefore, we first provide a general overview of acoustic features and then state how they were extracted.

*• line 488: Please explain the abbreviation CCF. What does the F stand for?*

We have added an explanation of this abbreviation. CCF stands for *cross-correlation function*.

*• line 496: given that the term “lag” is also used in the figures, it makes sense to explain shortly what is meant by this term here. What exactly is “lag zero”? Please explain. This can be done rather easily in intuitive terms as the concept is not so difficult to explain.*

We added a short explanation of the term lag right after it is introduced. We have also added a short explanation to the figures:

“We focused on cross-correlations at lag zero: A high cross-correlation between two scales at this lag means that both ratings change concurrently in the same direction as the music unfolds.“ (p. 23)

*• line 506: why the F0 in the CCFO?*

We have added an explanation that this refers to the cross-correlation function at lag 0:

“As predicted in H1, feeling moved or touched cross-correlated highly with experiencing warmth in the chest with an average of a cross-correlation function at lag 0 (CCF_0_) of .56 [.32, .80] across all excerpts.” (p. 23)

*• line 559: what does the B score refer to? Please explain. Same remark about sophistication of statistical analysis which may go beyond the competence level of the common readership.*

This refers to an unstandardized regression coefficient, B. This is a common notation in a regression context similar as a F-, t-, or p-value. We have added a short description in the text:

“Higher familiarity with an excerpt increased the liking of that excerpt with an unstandardized coefficient of B = -.42 [-.53, -.32], t(2380) = -8.05, p < .001.” (p. 15)

*• lines 573 ff: this is very technical section and very difficult to understand for a common reader. It is also very difficult to relate the text and the figure. Please explain much better how to interpret figure 4.*

We have included a description of the acoustic features discussed in this section, this should make understanding the paragraph easier.

*• line 589: same remark about statistical technicality: what does the rs stand for, why three values? Explain a little.*

In this case an *r* represents a Pearson correlation coefficient. The *s* mainly stands for the fact that these are several correlation coefficients that is also why there are three values. We have revised this section so we do present only one estimate and not three for each relationship.

*• line 604: same remark: Ward’s minimum variance method. Explain a little.*

This paragraph has been moved to the Supplementary Material. We have added a short description of this method there:

“Ward’s method, which at each step merges the two clusters leading to the least increase in the within-cluster sum of squares distance (between the points in a cluster to its centroid) was used to obtain the structure, [...]” (Supplementary Material 2.3)

*• line 630: it seems that, after having read the whole paper, very little bas been told about physiological sensations, besides feeling warm in the chest, and it can be asked whether this is a physiological sensation or simple a general bodily feeling. Perhaps “physiological and bodily reactions” may be a better term.*

*• line 665: same remark*

We agree with the reviewer and have included ‘bodily’ in the description:

“The purpose of this study was to explore continuous ratings of feeling moved or touched by music, and how the time course of feeling moved might covary with specific appraisals, emotional perceptions, and bodily sensations.” (p. 27)

*Figures and figure captions:*

*• Figure 1. Please explain used abbreviations: RE = random-effects? Q = ?: I = ?. The concept of zero lag should also be explained a little so as to make sense for the non-informed reader. A short intuitive description can suffice, either in the caption or in the main text.*

As mentioned above, we have included a description of lag 0 in the main text (and also added some sentences to the figure caption). We have also included descriptions of the abbreviation RE. Q and I2 refer to heterogeneity statistics:

“*Figure 2*. Cross-correlation functions (CCF; at lag 0, meaning that both time series are compared at the same time) between feeling moved and touched and the six main variables across the musical excerpts and sadly and joyfully moving songs separately employing the cubic spline detrending algorithm. Overall estimates and confidence intervals are constructed employing a random-effects meta-analysis using restricted maximum likelihood estimation. Error bars represent 95% confidence intervals. RE = random effects. As heterogeneity measures we included Cochran’s Q and I^2^.” (Figure 2 caption).

*• Figure 2. What are the units for the ratings? Please explain in the figure caption. This is done in the supplementary material but not here. Explain the seven point scale shortly.*

*• Figure 3. Same remark.*

We have added a description of the rating scales to the figures, e.g.:

“*Figure 3*. The averaged non-detrended continuous ratings of *feeling moved or touched*, *perceived beauty*, and *feeling a sense of connection* for the sadly (upper row) and joyfully moving (lower row) music excerpts. Ratings were provided on a 5-point scale (ranging from 1 (not at all) to 5 (extremely)) and aggregated in 3s time bins.” (Figure 3 caption)

*• Figure 4. Explain abbreviations. EC = Empathic Concern? It is difficult to relate the text of the figure caption with the figure. Very difficult to understand. Where are the p-values (*, **, ***) in the figure?*

We think that the reviewer is maybe referring to a different figure? In the original Figure 4 there was no reference to EC, this was done in Figure 5. We have provided more details on the abbreviation there (see Supplementary Material Figure S9). The p-values are presented in the original Figure 4.

• Figure 5: Same remark about intuitive understanding of the figure. How to find and visualize the four clusters. Please provide at least some intuitive explanation. Very technical matter. Difficult to understand.

There was an error in the numbering for the Figures 4 and 5 in the previous draft which has caused a misunderstanding. This error has now been fixed. In addition, we have moved the clustering analysis to the Supplementary Material as we think that it was not focal to our main research goals.

Suggested additional references

Juslin, P. & P.Laukka (2004). Expression, Perception, and Induction of Musical Emotions: A Review and a Questionnaire Study of Everday Listening. Journal of New Music Research, 33 (3), 217-238.

Reybrouck, M., Eerola, T. (2017). Music and its inductive power: a psychobiological and evolutionary approach to musical emotions. Frontiers in Psychology, 8, Art.No. 494. Open Access

We thank the reviewer for the additional sources. In fact, the original manuscript already included Juslin and Laukka (2004), as we agree that this is a highly important reference.

**Reviewer #3**

*This is an interesting study on the correlates of musically elicited states of being moved. Following previous work on this emotional state, the authors preselected their stimuli by either being joyfully or sadly moving, which was confirmed by the continuous ratings provided by the sample. The findings are mostly congruent to the expectations of the authors formulated prior to the study.*

*The manuscript is well organized and well written. With all this said, I would like to raise a number of theoretical and methodological concerns I see with this work, as well as some minor points.*

*1. Certain aspects, hypotheses and ideas seem to be not correctly attributed/referenced to the previous literature. For instance, in describing the rating paradigm of the present study, i.e. the continuous self-report, the authors state (line 267) “we aim to investigate experiences of feeling moved in response to music using a paradigm recently employed for studying kama muta evoked by short film clips (Schubert, Zickfeld, Seibt, & Fiske, 2016). Instead of prompting participants to rate their emotional reactions after watching a video, Schubert and colleagues instructed 909 participants to watch a number of different videos online (from a total number of 6) and rate their emotional reactions continuously while watching them.”. The continuous self-report has a long tradition in experimental psychology, reaching back at least to the 1940s (Peterman 1940), a great number of studies and several reviews has been published on that since then (for a recent overview, please see Wagner, Scharinger, Knoop, Menninghaus 2020). The impression of a novel design employed by this study (or that of 2016) is misleading. Similarly, the connection between chills/piloerection and being moved has been first proposed by Konecni (2005) and picked up by Benedek & Kaernbach (2011). First direct empirical evidence was presented by Wassiliwizky et al. (2015).*

Our goal was not to suggest that the current method or the design by Schubert et al. (2018) represent a novel paradigm. We mainly focused strongly on Schubert et al. (2018) as we built the present study (including scripts and materials) on their design. We have now reduced the space that is devoted to the discussion of this previous study and have mentioned that our study follows previous research using continuous self-report paradigms (citing the overview and additional studies that the reviewer suggested). We think that this addition reduces the impression of a novel design, which was not intended:

“In the present study we aim to investigate experiences labeled as feeling moved in response to music using continuous self-reports, a common paradigm in research on music and emotions that allows to model listeners’ responses to music dynamically (see Ruef & Levenson, 2007; E. Schubert, 2010; Wagner et al., 2020).” (p. 11)

Our overview on the relationship between chills/piloerection and feeling moved reads:

“Empirical studies have linked the occurrence of chills to strong experiences of feeling moved using qualitative methods, self-report ratings and more observational techniques such as a camera device (Bannister, 2018; Benedek & Kaernbach, 2011; Wassiliwizky et al., 2015; Wassiliwizky, Jacobsen, et al., 2017; Zickfeld, Schubert, Seibt, Blomster, et al., 2019). ” (p. 9)

We are not really sure at which point this sentence undermines the fact that some researchers have studied this relationship before others (in fact, the year of publication seems to be a good indicator for that aspect). The reference to Konecni (2005) is not included here as we only focus on empirical studies (and not theoretical articles). In fact, Konecni (2005) and the aesthetic trinity is introduced earlier in the section focusing on theories feeling moved.

*2. Menninghaus and colleagues’ position on being moved is mentioned repeatedly in the manuscript. However, one of their main points conceptualizing being moved as a mixed emotional state, which is overall always positive, but also includes negative emotions such as sadness, goes unmentioned. Physiological evidence for this position (contraction of corrugator in highly pleasurable moments), was first provided by Wassiliwizky, Koelsch, Wagner, Jacobsen, Menninghaus (2017) and later confirmed by Kimura, Haramizu, Sanada, Oshida (2019). The absence of this aspect in the manuscript is critical, because several result patterns are fully in line with this proposal: for instance, the cross-correlation between being moved and perceived happiness for *both* the sadly and the joyfully moving musical excerpts: CCF = 0.49 [.08, .90] and CCF = 0.61 [.48, .75], respectively. (Overall CCF = 0.67 [.59, .74]). Also note that the happiness and sadness time series in Figure 3 fluctuate in line with the mixed affect proposal. In Wassiliwizky et al. (2015), for instance, the following formula is proposed: For being sadly moved, sadness is in the foreground, but happiness still in the background, whereas for being joyfully moved the reversed picture is true with happiness in the foreground and sadness in the background. Exactly this is reflected by the time courses in Figure 4. Sadness is not 0 (or 1 in this case) for the joyfully moving pieces and reversely happiness is not 0 (or 1 in this case) for the sadly moving pieces.*

*See also the conclusion in line 684: “When the overall emotional tone of the music was sad rather than joyful, feeling moved or touched cross-correlated with both perceived sadness and perceived joy” These effects appear even for a small number of stimuli and despite the potential bias in the stimulus choice (see point 8 below). Given this situation, I think the interpretation of being moved as a mixed affective state should be offered and discussed in the manuscript.*

We have added to our theoretical discussion that feeling moved is considered a mixed emotional state by Menninghaus and colleagues. As the reviewer argues, our findings are mainly in line with this theoretical position and we have added a paragraph to the Discussion to acknowledge the perspective that feeling moved might represent a mixed emotional response:

“First, it could mean that, although predominantly positive, feeling moved constitutes a mixed state including both negative and positive affect, as suggested by one specific theory (Menninghaus et al., 2015). ” (p. 30)

*3. In referring to the literature that links musical chills to the psychoacoustic/musical parameters (p.10/11) the authors might want to add the recent study by Bannister (2020) on the looming effect.*

We thank the reviewer for this important reference and have added it to the manuscript:

“Relatedly, an experimental study found that increased loudness (acoustic intensity) and reduced brightness (proportion of high to low frequencies) resulted in more frequent reports of chills (Bannister, 2020b). ” (p. 10)

*4. The third a priori hypothesis connects being moved to sadness for the sadly moving pieces (line 334: “the time course of feeling moved correlates positively with the time course of perceiving the music as sad for sadly moving excerpts, while it does less so for happily moving excerpts”). How does this fit to the results from 2016 discussed earlier? i.e., “the time series results showed no or even negative associations between these two variables [feeling moved and feeling sad] for most videos (line 280)”.*

As mentioned earlier, we have reduced the discussion of Schubert et al. (2018). In addition, this hypothesis fits as we focus on the distinction of *sadly* and *joyfully* moving music. As mentioned by Menninghaus et al. (2017), Schubert and colleagues (2018) focused mainly on *joyfully moving* videos (although there was some variance in the cross-correlation between feeling moved and sadness). In addition, Schubert et al. (2018) asked about how happy or sad the participants felt while watching the videos, whereas in the present study, we asked how joyful or sad the music sounded. This is an important distinction, as expressed sadness does not necessarily evoke sadness in the perceiver, but can also lead to anger, schadenfreude, kama muta, guilt or other emotional responses, depending on the context (although previous studies suggest that experienced and perceived sadness/joy are highly correlated, Vuoskoski & Eerola, 2017).

*5. Another question regarding the same third hypothesis, linking being moved to sadness, is: How does this hypothesis relate to the conceptualization of kama muta as a purely positive emotional state (line 148: “kama muta [...] is experienced as primarily positive”)? Why is kama muta, being a positive emotional experience and probed by the label “being moved”, connected to experiences of sadness?*

We have provided a short discussion why kama muta is related to negative emotions (and especially the negative of emotion often referred to as sadness) in the Introduction and a more detailed treatment in the Discussion section. First, we define an emotion by the co-occurrence of different emotion components (see Lange et al., 2019). Kama muta is argued to occur when the different appraisal components, feeling components, physiological components, expressive components, and motivational components associated with this emotion co-occur (though there is limited evidence on which one of these is necessary to occur). In its definition, the feeling component of kama muta is positive (e.g., Fiske et al., 2017). However, as no emotion occurs in a vacuum kama muta can co-occur with other emotional experiences, some of which can be negative in their valence. A frequent emotion that shows overlap with kama muta, is the emotion typically referred to as sadness. They can overlap because they share emotion components with each other (see Lange & Zickfeld, 2021). In the case of kama muta and sadness, there is for example overlap in the physiological component related to tears, but also in the appraisal component. Kama muta theory thus argues that the emotion of kama muta can co-occur with sadness. In addition, communal sharing often intensifies when people open up to each other and share their emotions. Music can be perceived as emotional communication. Thus, listening to sad music can be a moment of great intimacy, tuning into another person’s expressed sadness, and thereby giving rise to kama muta. Specifically we write:

“Finally, we had hypothesized that feeling moved or touched would cross-correlate with perceived joy for joyfully moving excerpts but less so for sadly moving excerpts, and with perceived sadness for sadly moving excerpts but less so for joyfully moving excerpts. While feeling moved cross-correlated with perceived joy, but not sadness for joyfully moving music, the picture regarding sadly moving music turned out to be somewhat more complex: When the overall emotional tone of the music was sad rather than joyful, feeling moved or touched cross-correlated with *both* perceived sadness and perceived joy (except for the excerpt “Oblivion”). The finding that ratings of feeling moved or touched cross-correlated positively with perceived joy across both joyfully and sadly moving excerpts, supports previous theories suggesting that the overall tonality of the experience of feeling moved is positive (Zickfeld et al., 2019). This is in fact reflected in most perspectives on feeling moved (cf. Bartsch et al., 2014). The finding that feeling moved or touched cross-correlated positively with perceived sadness for sadly moving excerpts can be interpreted from two different angles. First, it could mean that, although predominantly positive, feeling moved constitutes a mixed state including both negative and positive affect, as suggested by one specific theory (Menninghaus et al., 2015). Second, this could mean that feeling moved is experienced as predominantly positive but can co-occur with other negative emotions such as sadness, as suggested by kama muta theory (Fiske et al., 2017). The observation that feeling moved or touched cross-correlated negatively with perceived sadness for joyfully moving excerpts provides some evidence for the latter explanation. However, it should be noted that we assess perceived and not experienced sadness and joy which means that our findings can only be interpreted as indirect evidence. In sum, the main findings support the idea that feeling moved is perceived as predominantly positive - also in the context of music.” (p. 30)

*6. It is important to state explicitly that we have a between-subject-design here; it took me quite a while to gain certainty on that due to the somewhat misleading descriptions, e.g. “Participants were presented with all seven excerpts and all seven scales”, line 398. This has far-reaching implications for the interpretation of the time series results. Specifically, we can never conclude that the same person experienced, for instance, being moved and chills at a time. Thus, all interpretations are to be seen on the group level. I see this as a limitation for the present study.*

We agree that the sentence on line 398 was somewhat misleading. We have restructured this part of the Method section and mention explicitly that our design is a 7x7 mixed design also highlighting that responses can only be compared on the group level. We agree that this represents a limitation. Importantly though, previous research suggests that within measurements in continuous self-report paradigms are rather uncommon (E. Schubert, 2012). For the present set-up it seemed unfeasible to have participants listen to the same musical excerpt seven times and provide different ratings. We think that our design can also be seen as a strength as it shows that ratings cross-correlate highly although they were never given by the same individual.

*7. Another limitation for me is the fact that participants were asked to provide ratings on perceived --and not felt-- emotions (“we targeted the emotions perceived in the music and not felt emotions”, line 305). Given the strong focus on the experiential aspects in the rest of the paper, I have difficulties to understand this decision. Could the authors provide reasons for their choice of task?*

We have added a short justification why we focused on perceived emotions in the case of happiness and sadness (note that the feeling moved or touched item focused on felt emotions). Our main goal was to reduce the conflation of participants' ratings of their own happiness with the degree to which they enjoyed the music. As mentioned in the manuscript, there is a high overlap between ratings of felt and perceived emotions in research on music. Nevertheless, we have added a short paragraph in the Limitation section in order to acknowledge the shortcomings of our decision to assess perceived rather than felt emotions:

“ Specifically, we targeted the emotions perceived in the music and not felt emotions for these two items, although it should be noted that the two processes are strongly related (see Vuoskoski & Eerola, 2017). This choice was made due to the possibility that the continuous ratings of experienced joy could be confounded by enjoyment of the music as a result of being moved by the music. We were, however, more interested in the elicitors of feeling moved or touched.” (p. 13)

*8. Another limitation to be discussed is the experimenter-selected choice of stimuli for this study. It is difficult to rule out the possibility that the experimenters were biased/guided by their concept of kama muta when selecting the stimuli. In the light of the small number of stimuli (see Limitations section) this might be a serious problem, particularly for the analyses on the psychoacoustic correlates, but also for connecting movingness in music to social cohesive effects. I strongly doubt the universality of this link. Symphonic work that has classically been labeled as intensely moving such as the first movements of Beethoven’s fifth symphony or Mahler’s second symphony are only anecdotal references to be mentioned here.*

We agree that experimenter-selected stimuli can include some limitations and we have added a sentence to the Limitation section. On the other hand, employing self-selected stimuli minimizes control and would not have allowed modeling dynamics for each specific song as in the present study. We think that a replication of the current findings using self-selected music would make a strong case:

“Future studies could investigate the contributing features in a larger and more varied set of music examples. A possibility for targeting such larger stimuli sets could be to instruct participants to self-select moving music (e.g., Mori & Iwanaga, 2017; Wassiliwizky et al, 2017). While such a design minimizes the control over the stimulus material, it would be interesting to see whether the current findings replicate when employing such a technique.” (p. 36)

*9. Relatedly, I am wondering why “two of the excerpts included lyrics” (line 435), since this study stresses the focus on the link between being moved and instrumental music. Although the languages might be foreign to the perceivers, they can still trigger associations and even more critically convey emotions by the voice. Why could this issue not have been circumvented during stimulus selection? From my perspective, this should at least be discussed as a limitation.*

We agree with the reviewer and have added a short acknowledgement to the Limitation section. In general, the effects for the two musical excerpts including lyrics did not differ substantially from the remaining pieces:

“We also included two musical excerpts that featured vocals. While none of the participants were proficient in the depicted languages, the human voice is known to transport specific emotions as well as empathy (e.g., Bowling et al., 2012). Nevertheless, the two vocal pieces showed similar results as the instrumental excerpts. ” (p. 37)

*10. It is unusual to have chills ratings as a continuous measure (line 415: “To what degree do you experience chills (goosebumps) right now?”). Usually, they are probed by a binary on-off signal (e.g. pushing a button for the time a chill is experienced). This should be pointed out in the text. Additionally, while I see the advantages of classifying chills as rather mild or strong by this approach, I am also somewhat concerned (at the lower end of the scale) that false positives have been facilitated here. To rule out this concern, I would ask the authors to report the average number/min and duration of chills in their study, i.e. average time stretches between 2/3/4/5 and 1 on their scale. Thus we could compare these values to previous studies that quantify chills/min and the duration of an average chill experience.*

We have added a short mention that some previous studies have used dichotomous rating scales for chills:

“Note, that some previous studies have assessed chills using a dichotomous measure probing whether participants experienced chills or not (e.g., Wassiwiliziky, Jacobsen et al, 2017). Based on T. W. Schubert et al. (2018), we were interested in differentiating various intensities of chill responses.“ (p. 18)

We have performed the analyses as suggested by the reviewer. Across all participants, ratings, and songs, 54.89% of the time a chill rating of 2 or higher was given, suggesting that participants experienced at least some mild form of chills. When looking at the average duration of chills, we found that the average duration of ratings 2 to 5 was 60.72s. Focusing on the specific scale points this differed between 15.62s and 21.20s for individual scale points. Results are presented in the Supplementary Material (2.2). These estimates may be somewhat conflated, however, since we were interested in correlations, and not in means, that should not have impacted the findings in any substantial way.

*11. Relatedly, musically induced chills have always been conceptualized to reflect peak emotional experiences. When we look at the average ratings in Table S3 we see rather low numbers, particularly for the chills themselves. To have a better impression on the local fluctuations I would like to see the continuous average ratings for the physiological variables (chills, warmth in the chest) presented in the same way as the emotional time series in Figures 2 and 3.*

We agree with the reviewer that this can be quite helpful and have added an additional Figure showing the relationship between feeling moved, chills, and warmth in the chest (Figure 4). As can be seen from this figure, ratings of chills varied for the different pieces. For example, for Explosions we can see that there is a change in the music for the last seconds when chills (and the other variables) show a peak.

*12. I sometimes missed the statistically informed justifications for handling the data a certain way in the Data Preparation section. For example, the authors binned the data into 3 seconds units. Why? They refer to their previous paper which used the same strategy. However, this is not a statistical or methodological reason (line 446: “Similar to Schubert and colleagues (2016), we decreased the resolution of the time scales by aggregating judgments within units of three consecutive seconds”).*

We have justified our decision to bin the data into 3 second units in more detail. Specifically, we write:

“Third, we decreased the resolution of the time scales by aggregating judgments within units of three consecutive seconds. We assumed that participants were not able to report their momentary experiences within the exact same second they appeared. Previous research has suggested that 3-seconds represent the temporal building blocks of many aspects related to human perceptual or motor abilities (Pöppel, 1997). Therefore, we chose three seconds as an adequate time bin (Figure 1, 3).” (p. 19-20)

*13. Similarly, out of three different variants of detrending, the authors decided to stick to the spline-detrended data for all further analyses, without providing any (statistical) reasons for this decision. As this deviates from the preregistered procedure, I think this decision should be substantiated.*

We agree with the reviewer that this deviation from our registered protocol should be substantiated more. We now write:

“Note, that we originally registered to employ the residual detrending method. However, cubic splines have been considered to be superior when it comes to removing slow drifts and have been recommended to handle time series data when collecting emotional responses (McKeown & Sneddon, 2013; Tanabe et al. 2002). Results of the two additional detrending methods are reported in the Supplementary Material. ” (p. 21)

*14. In general, detrending time-series-ratings is reasonable given the effects mentioned in the paper, which would artificially inflate the cross-correlations (i.e., general rating behavior of participants leading to quadratic and cubic trends). However, in such detrending procedures only local/rapid changes survive, whereas long-term trajectories are filtered out and remain unattended. This might be problematic for the rather sluggish signals we are dealing with here (affective fluctuations). Particularly the detrending of chills experiences seems quite counterintuitive to me. I would ask the authors to discuss this issue and also to provide figures with the detrended time series data (in the Supplementary Material), similarly to Figures 2 and 3. At present, the time series we see in Figures 2 and 3 represent raw data that was not the basis for any further analysis.*

We have added a short discussion on the effects of detrending on our time series data:

“The phenomenon of feeling moved tends to happen over shorter-time intervals and is not expected to demonstrate long-term trajectories, as typically assumed for emotional experiences (in contrast to longer-lasting moods; Beedie et al., 2005). Hence for the purpose of this study detrending does not affect this significantly. Especially, the cubic spline detrending that we employ ensures that we capture these shorter-term fluctuations while removing the long-term trajectories which are more an artefact resulting from the non-stationary nature of the emotion ratings (e.g., Bringmann et al., 2018).” (p. 21)

In addition, we now provide the detrended figures in the Supplementary Material (Figures S6-8), as suggested by the reviewer.

*15. Why only US citizens have been allowed to participate?*

We only requested US American participants, as emotional responses to music might be influenced by different cultural backgrounds. As there are many Indian participants in the workerspool on Amazon MTurk, we applied this criterion. We now explicitly write:

“We excluded non-US participants to ensure that the participants had a shared, consistent understanding of the emotional labels and concepts used in the rating tasks, and to control for possible effects of cultural background on emotional experiences in response to music (cf. Laukka et al., 2013). “ (p. 16)

*16. In justifying the sample size, the authors state (line 364): “Based on recommendations by Schubert, Zickfeld, Seibt, and Fiske (2016) we planned on collecting 40-50 participants per cell”. It is somewhat misleading here to refer to another empirical study and not to statistical methodological work. BTW: In Schubert et al. the sample size of N=40 is already referred to as a previous recommendation, without, however, giving a reference.*

We agree with the reviewer that it can be misleading and should be avoided as a heuristic to refer to previous empirical studies for sample size justifications (see Lakens, 2021). However, as we have originally registered our sample size based on these criteria we mention them for transparency. We have added some reference to previous studies using continuous self-report paradigms and have conducted a simulation to assess the precision of our own data based on McKeown & Sneddon (2013). Based on these aspects, we think that the original planned sample size of 40-50 participants per cell is sufficiently powered to detect the present effects:

“Previous studies employing continuous self-report paradigms have not considered sample size justifications systematically (E. Schubert, 2010; Ruef & Levenson, 2007; Wagner et al., 2020). As an exception, a recent methodological paper considered between 20 and 30 raters per cell (combination of independent variables) to be sufficient, though this number was dependent on the specific study design (McKeown & Sneddon, 2013). We originally registered to collect 40-50 participants per cell based on a previous study using a similar procedure (T. W. Schubert et al., 2018). Performing a post-hoc precision analysis on our data following McKeown & Sneddon (2013) suggested that this number was sufficient (Supplementary Material Figure S5).” (p. 15)

*17. The assignment of Figures 4 and 5 is mixed up in the text (e.g. line 617: “The averaged feeling moved or touched ratings for each of the clusters can be seen in Figure 5” should be “Figure 4”).*

We thank the reviewer for pointing this out. As we have added additional figures numbering has changed so that the cluster graph is not longer Figure 4. In addition, we have moved the cluster analysis and its graph to the Supplemental Material (now Figure S9).

*18. The results of the clustering analysis in Figure 4 seem a little bit lost, because the effects could be shown only for two stimuli. I would therefore recommend to interpret them very carefully (currently, we read in line 781 quite generally that empathic concern is associated with more pronounced peaks in continuous feeling moved ratings).*

We agree with the reviewer that the results of the cluster analysis should not be overgeneralized as they only observed effects for two musical excerpts. We have now moved the majority of the cluster analysis to the Supplementary Material (2.3), as we think that the analysis is not focal to the main goal of our manuscript. We briefly mention the results in the Discussion section, but highlight that the findings should be interpreted with caution, as they were only observed for two songs.

*19. The conclusion in line 785 (“these results give support to the role of empathic processes in emotional responses to music (cf. Miu & Vuoskoski, 2017), emphasizing the inherently social nature of musical experience”) seems overgeneralized and overstated given the fact that only moving music has been employed here and the fact that a major study on the functions of music by Schaefer et al. (2013, cited in the manuscript) has identified expression of social relatedness only as a subordinate factor. Ethnographical work on war music and music to intimidate the adversary are further counterexamples to mention here.*

We agree with the reviewer that this conclusion was overgeneralized and we have revised it accordingly. We now state:

“The current results suggest an important role of empathic processes in emotional responses to moving music (see Miu & Vuoskoski, 2017; Huron & Vuoskoski, 2020), and are in line with the view of musical experience as inherently social (e.g., Savage et al., 2020). Previous studies have argued that empathic engagement with music could take the form of resonating with the expressions and imagined experiences of the performer or the composer (e.g., Scherer & Zentner, 2001), or identifying with an imagined narrative or a virtual persona represented by the music (Levinson, 2006). ” (p. 34-35)

*20. What are the error bars in Figures 2 and 3?*

These error bars originally referred to the standard error of the averaged individual ratings. For the purpose of clarity we have removed the error bars in the revised manuscript.

**Reviewer #4**

*This article describes a thorough and competent piece of research investigating a popular topic in the field of musical emotions (and aesthetics more generally). Nonetheless, there are a few points that I think deserve the authors attention some of which have implications to the interpretation of the results. I hope that these comments help to improve the manuscript.*

*1. The manuscript in general, and the introduction in particular, could be streamlined. The introduction lacks fluidity because there is a tendency to concatenate the various ideas, concepts, and arguments without carefully crafting the key message. Also, the results description is rather “thick” and could be simplified if the authors focus on the key findings without overinterpreting or trying to explain to much about smaller (sometimes irrelevant) results.*

We have restructured the Introduction and attempted to streamline it. We now clearly focus on feeling moved in the context of music and its theoretical components, as well as acoustical correlates. In addition, we have simplified that Results section by removing reference to individual effects and mainly focusing on overall effects. We have also tried to focus on the key findings and interpret findings based on their boundary conditions. For example, we have moved most aspects of the cluster analysis to the Supplementary Material, as we realized that it is not focal to the main goal of the manuscript.

*2. Line 165: in the introduction perhaps it is not so relevant to include statistical details, especially because the same is not done for other works as well.*

We have restructured this section and have removed the statistical details as suggested by the reviewer.

*3. The section starting in line 173 would benefit from a description of the work by Coutinho and Scherer (Coutinho, E., & Scherer, K. R. (2017). Introducing the GEneva Music-Induced Affect Checklist (GEMIAC) A Brief Instrument for the Rapid Assessment of Musically Induced Emotions. Music Perception: An Interdisciplinary Journal, 34(4), 371-386.). They did a comparison between the meaning of emotions commonly use to described musical emotion and feeling moved/touched is part of it. What is particularly relevant about their work is that they established the proximity of these feelings to other feelings of emotion, which particularly relevant for this work and perhaps more informative that the sources already cited.*

We thank you for this suggestion. We have added a short paragraph explaining the findings by Coutinho and Scherer (2017) with respect to feeling moved:

“Items assessing feeling moved have been included in the Geneva Emotional Music Scale (Zentner et al., 2008) and a hierarchical clustering approach suggests that feeling moved (as measured by the items moved and touched) form a distinct cluster of musically induced emotions (Coutinho & Scherer, 2017). In this study feeling moved was most similar to the states of wonder and transcendence.“ (p. 7)

*4. Paragraph starting in line 260: First sentence should be further developed to justify the statement. Last sentence also needs a justification (the authors say that they argue the idea but do not present arguments).*

We have revised this paragraph now justifying our statements:

“ Not only music performance but also music perception seems to be inherently social. Listeners can infer affiliative motives from musical improvisations (Aucoturier & Canonne, 2017), feel connected to music through a form of empathy (Van den Tol & Edwards, 2015), and music can increase connectedness towards aspects conveyed and embodied by music (Vuoskoski et al., 2017). ” (p. 10)

*5. Line 279: There is evidence that summative and continuous ratings are different things, and the average of continuous ratings does not correspond to summative ones. Therefore, the statement between brackets is not correct. I suggest that the authors develop their ideas in this respect in this part of the manuscript.*

We have restructured this paragraph and removed the aspect the reviewer was referring to. Now, we do not imply that summative and continuous ratings are the same.

*6. Lines 305-7: emotions felt and perceived are different. They can be the same, but that also depends on many variables (some of them quite contextual and transient). My point is that the justification presented to used perceived emotions is not clear and needs careful consideration. One may argue that it does not make sense at all compare feeling moved with perceived joy or sadness. In fact, this is a weakness of this work. I suggest that the authors provide stronger rationale for option for perceived emotion ratings and explain in the conclusions that this is acknowledged as a potential limitation in the discussion section.*

We have added a short justification why we focused on perceived emotions in the case of happiness and sadness. Our main goal was to reduce the conflation of participants' ratings of their own happiness with the degree to which they enjoyed the music:

“Specifically, we targeted the emotions perceived in the music and not felt emotions for these two items, although it should be noted that the two processes are strongly related (see Vuoskoski & Eerola, 2017). This choice was made due to the possibility that the continuous ratings of experienced joy could be confounded by enjoyment of the music as a result of being moved by the music. We were, however, more interested in the elicitors of feeling moved or touched.” (p. 13)

As mentioned in the manuscript, there is a high overlap between ratings of felt and perceived emotions in research on music. Nevertheless, we have added a short paragraph in the Limitation section in order to acknowledge the shortcomings of our decision to assess perceived rather than felt emotions:

“Finally, we assessed perceived joy and sadness in the music, and not experienced joy and sadness induced by the music. Previous studies (Vuoskoski & Eerola, 2017) and our pilot study suggested that ratings of perceived and experienced emotions in music correlate highly. Nonetheless, it should be emphasized that our measures only allow for indirect inferences about participants’ experiences of joy and sadness.” (p. 37)

*7. Lines 330-228: text can be simplified. All hypothesis chare the same start. Also, hypotheses III and IV are expressed in a strange way and they seem to overlap. I suggest that you remove the text after the commas in both.*

We have simplified our hypothesis section according to the reviewer’s suggestion. We have kept hypotheses III and IV for two main reasons. First, these represented independent hypotheses as one concerns ratings of perceived sadness and the other ratings of perceived happiness. Second, in the pre-registration hypotheses were listed as they are now. Thus, we decided to keep them as they are.

*8. Line 419: what are the values between brackets?*

Numbers in brackets refer to the value of the rating scale. We have now clarified this aspect in the manuscript.

*9. You collected data that does not seem to have been used in the analysis, especially relating to demographics. Is there a specific reason for that? Could it be that, like trait empathy, these variables could explain differences between subjects? Note as well as there are well established measures of musical proficiency that should have been used.*

We did not focus on other demographic aspects, as previous research on feeling moved has revealed rather little systematic differences in the experience for variables such as gender or age (e.g., Zickfeld et al., 2019 for a review). Our data are openly available online so other researchers could test these aspects and generate hypotheses based on them. However, we think that these tests are beyond the scope of the current manuscript.

We agree with the reviewer that there exist validated measures of musical proficiency (e.g., Zhang & Schubert, 2019) that we should have used in our study.

*10. Data preparation: participants continuous ratings are detrended because the of the start and end segments trends. Nonetheless, the authors end up removing the start of the segments which was actually the initial justification for detrending the data. This seems somewhat contradictory. Also, excessive data processing can be very negative as it also eliminates relevant information that you may not be aware of. Intuitively, if the authors are interested on ratings dynamics, any pre-processing that affects that dynamics can have a direct impact in the findings. Can the author explain if using the original data without the start and end segments (which indeed are very much affected by the transition from/to music to/from silence) leads to the same results and conclusions presented in the paper? In my view the paper should report the findings without detrending the data. Unless I am missing something, in which case I would ask the authors to justify more clearly the need for detrending and the consequence that it has on the results and conclusions.*

We think that this might represent a misinterpretation of our procedure. We detrended ratings in order to remove the trend in our time series to make them stationary:

“Fourth, we detrended the time series. Most time series are non-stationary, meaning that they show linear or higher-order increases or decreases. Comparing two non-stationary time series would inflate correlations, as they tend to cross-correlate simply due to the fact that they have trends in common. Detrending, removing the trend in time series, is one possible way to achieve the stationarity of time series data (Wu et al., 2007; Shumway & Stoffer, 2011). Inspecting our time series, all averaged curves showed increases in ratings over time and some also decreases in ratings towards the end. More specifically, they indicated positive linear and negative quadratic trends. We detrended all time series using a cubic spline interpolation (Wu et al. 2007; McKeown & Sneddon, 2013). “ (p. 20)

Detrending was not chosen because of the start or end segments as suggested by the reviewer. We decided on removing the first three bins from each time series because we expect that these ratings could be confounded by the fact that music starts from silence and not so much reflecting actual changes within the music. E. Schubert (2012) has referred to this as *initial orientation time* and suggested a median of 8s for this process to settle. Therefore, we have decided to exclude the first three bins (9s) for each segment.

We were not really sure what the reviewer meant with “can the authors explain if using the original data without the start and end segments [...] leads to the same results [...]?”. We were not sure what the reviewer meant with ‘end segments’ in this case and how that should be defined. Many of our excerpts included fade outs and we don’t see any reason why specific end segments could influence participants’ ratings and therefore should be removed.

We present the undetrended results in the Supplementary Material. As mentioned in the main manuscript, the direction of these effects is similar, though effects are much stronger. We argue that this represents an overestimation due to underlying trends in our time series that is why we apply detrending to our data. We have justified this decision now more clearly.

*11. Also pertaining to the same point, there seems to be the tendency to use data-processing strategies from another article without further scrutiny. In addition to potentials limitations, it has also led to some inconsistencies. One of them is the fact that the authors ” we decreased the resolution of the time scales by 448 aggregating judgments within units of three consecutive seconds” but used a sliding window (moving average) for acoustic features. This is inconsistent.*

Acoustic features were extracted using standard techniques. Sliding-window analysis is the norm in the field of Music Information Retrieval (MIR). The sliding-window allows us to look at how the feature varies smoothly in time versus a non-sliding-window approach which may lead to abrupt changes in the feature. Also, there are typically short-term features that are extracted using an analysis window in the order of milliseconds (Tzanetakis and Cook, 2002; Aucouturier & Pachet, 2003; McAdams, Winsberg, de Soete, & Krimphoff, 1995), and long-term features that are context-dependent and are extracted using an analysis window in the order of a few seconds. This long-term window length was chosen because it corresponds to typical estimates of the length of the auditory sensory memory (Fraisse, 1982) which is required to get a sense of context to calculate the higher-order features (which uses the same length as the self-report ratings). This approach has already been used in previous time series studies (Alluri et al. 2012; Burunat et al. 2016).

For the self-report ratings, we created time bins of 3 seconds as done in previous research (Schubert et al., 2018) based on findings that this represents the temporal building blocks of perceptual ratings (e.g., Pöppel, 1997). In order to match the self-report ratings we finally resampled the acoustic features so that they were also presented by a 3-second resolution. We realized that this final step was not clear from the manuscript and might have led to the idea that the processing steps of the acoustical features and self-report ratings were inconsistent. We have added a sentence to make this clear:

“Short-term features were extracted using a 50ms window with a 50% overlap and encompassed loudness and timbral properties. While loudness was captured by the feature root-mean-square energy, timbral features primarily comprised spectral shape descriptors, namely spectral centroid, spectral spread, spectral roll-off, entropy, roughness, and flatness. In addition, spectrotemporal fluctuations were captured by spectral flux and temporal fluctuations by zero-crossing rate. Long-term features capture context-dependent tonal and rhythmic aspects of music and are extracted using a three second window with an overlap of 67%. Tonal variation was captured by key strength or key clarity while rhythmic variation was captured by pulse clarity. All features were extracted from each excerpt using the MIRtoolbox (Lartillot & Toiviainen, 2007). Subsequently, spline detrending was performed on the acoustic features, and the first 9 seconds of the excerpt were excluded from analyses as done for the emotional ratings. Finally, we created time bins of three seconds in order to match the self-report ratings.” (p. 22)

*12. Can the authors explain why acoustic features were also detrended and what the impact on the data was?*

This operation was performed to eliminate those low-frequency components whose eventual perceptual correlates were eliminated during the detrending stage of continuous self-report ratings. In fact, this is a necessary operation to ensure that both time-series are comparable. When detrending the self-report ratings we do not only remove the slow or global drift that occurs due to the nature of the data but also the slow drift that might occur due to slow changes in acoustic features. The same detrending process for the acoustic features is therefore necessary, since we already removed potential slow drift correlates from the self-report time series.

We have added a sentence to acknowledge this fact:

“Subsequently, spline detrending was performed on the acoustic features, and the first 9 seconds of the excerpt were excluded from analyses as done for the emotional ratings. Finally, we created time bins of three seconds in order to match the self-report ratings. Detrending of the acoustic features was necessary to ensure comparability with the detrended self-report time series, which removed potential slow drift or long-term features. “ (p. 22)

*13. Note that figures are of very small quality (at least in the document I receive) and I could not use them as supporting material as I could not read the captions and legends.*

We apologize for the low quality of the original figures and have now revised them.

*14. There is a tendency to overinterpret the results, which sometimes leads to taking conclusions from the analysis of a single piece. A limitation of the study is that it is based on correlations and only a small number of pieces (I perfectly understand that there were reasons to focus only on a few number of pieces) and over interpreting the data can mislead the reader regarding the importance of the findings.*

We have revised our discussion focusing on the issue of overinterpretation and removed statements that were too general. We also address the aspect that we use a small number of pieces in the Limitation section. Specifically we write:

“An obvious limitation of the present study is that only seven music excerpts were used as stimuli. However, this aspect of the experiment design was closely tied to the constraints of the continuous rating paradigm. This limitation is particularly relevant for the analysis of the acoustic and musical features contributing to feeling moved, since the pattern of results may be specific to the particular music excerpts. It is likely that the acoustic and musical characteristics associated with feeling moved vary somewhat from piece to piece, and thus our findings reflect only a part of the entire picture. Moreover, the musical features that were explored in the present study do not represent an exhaustive selection of possible musical features, and thus there may be other, additional musical or acoustic features that contribute to feeling moved or touched. It is also likely that the relationship between musical features and perceptual ratings is non-linear, limiting the explanatory power of linear analysis methods such as those used in the present study. Similarly, we observed higher heterogeneity in effects for the *sadly moving* excerpts in comparison to the *happily moving* excerpts. This was mostly driven by *Oblivion* that typically showed smaller effects than the other two *sadly moving* excerpts, but sometimes even the opposite as for example for the relationship between feeling moved or touched and warmth in the chest. It is unclear whether that particular excerpt represents a more prototypical or more atypical version of the category of *sadly moving* music. Future studies employing a larger stimulus pool could remedy this shortcoming.

” (p. 35-36)

*15. I also think that there are interesting trends in the data that are not captured by the correlation analysis. I wonder what the findings would be if the authors would compute the correlations on time series using larger bins (more than 3 seconds) and using overlapping windows to avoid sharp (artificial) changes.*

This certainly would be interesting to do. However, we believe this merits its own thorough methodological investigation which we believe is beyond the scope of this paper. Moreover, a 3 second long bin is comparable to auditory sensory memory (Fraisse 1982; Pöppel 1997) which makes it a reasonable choice as a tradeoff between specificity and interpretability. We now include a short paragraph to reflect on the choice of using 3-second bins:

“We assumed that participants were not able to report their momentary experiences within the exact same second they appeared. Previous research has suggested that 3-seconds represent the temporal building blocks of many aspects related to human perceptual or motor abilities (Pöppel, 1997). Therefore, we chose three seconds as an adequate time bin (Figure 1, 3). “ (p. 20)

16. Line 564: why did the author use Spearman rank correlation coefficient for acoustic features data and (I suppose) Person’s correlations coefficient for the rest of the data?

We employed cross-correlation functions for the main analyses. As we did focus on cross-correlations at lag 0 these indeed reflect a zero-order Pearson correlation coefficient.

We used Spearman correlation for acoustic features since the range of acoustic features was variable and distribution might represent a problem. This was considered a smaller problem when it came to the self-report ratings as they were fixed on a 5-point Likert scale (and potential *influential points* are less likely). We repeated the main analyses comparing the self-reported time series using Spearman rank correlation coefficients, which is presented in the Supplementary Material (Figure S9). In general, coefficients and relationships were similar to the results presented in the main manuscript.

*17. I could not understand exactly how the authors performed the clustering analysis (line 594 onwards) using the time-series data and why they chose the described the method. Wouldn’t it be possible to use functional ANOVA for this or other well established and robust methods?*

We have now added more details about the clustering. Also the main reason for performing agglomerative clustering since it is a well-established approach to identify inherent data-driven clusters or groups. We perform robust well-established non-parametric statistical tests owing to the nature of the data. We further explain our choice and hope that it clarifies this concern. In general, we have moved the cluster analysis to the Supplementary Material (2.3), as we think that this streamlines our Result section and puts the focus on our most important findings.

18. Results pertaining acoustic features are overinterpreted. The authors neds to keep in mind that there are only 7 tracks and some points being made only pertain to a single track (and the interpretation of the feature meaning is rather subjective). Section starting in line 711 should reflect these changes (as well as in the conclusions). Another important thing that I suggest the authors consider in their interpretation is the fact that correlations between acoustic features and feeling moved may simply be the result of these ratings being correlated with joy or sadness, i.e., if being moved is correlated with perceived joy and perceived joy is correlated with feature X, than we can expect a correlation between being moved and feature X. Nonetheless, we cannot conclude that this correlated in meaningful or that it is not mediated by joy. Correlations must be interpreted carefully.

We thank the reviewer for highlighting this point. We agree that the kind of emotion indeed might mediate the response of feeling moved which in turn would be reflected in the acoustic features. We have added a paragraph in the Discussion that addresses these issues:

“Importantly, the current findings are based on a small number of musical excerpts so caution should be applied when interpreting these findings, especially the inconsistent results for sadly moving excerpts. In addition, since continuous ratings of feeling moved cross-correlated strongly with perceived joy or perceived beauty it is possible that the observed acoustic features are not specific to feeling moved, but rather reflect co-occurrences with other evaluations or experiences. Altogether, we observed correlational evidence that can guide future studies in experimentally different musical aspects and measuring ratings of feeling moved (e.g., Bannister, 2020).” (p. 33)

*19. Generally, the discussion and conclusions could be slightly toned down to avoid over interpreting the results of the study.*

We agree with the reviewer that parts of the Discussion can be toned down to avoid overgeneralizations. We have revisited different paragraphs and now explicitly discuss the boundaries of our findings. For example, as described in response to the previous comment we have highlighted that the findings pertaining to the acoustical features could be based on different variables. Similarly, we have toned down the discussion related to the findings of the trait empathy measures. As we have moved the cluster analysis to the Supplement, we now explicitly highlight the fact that effects were only observed for a limited number of stimuli and that these findings should be interpreted accordingly.

When talking about the importance of empathy in the experience of music we for example now write that “the current results suggest an important role of empathic processes”, instead than writing that “these results give support to the role of empathic processes in emotional responses to music” (p. 34).
