# Supplementary material for: Feeling moved by music: Investigating continuous ratings and acoustic correlates
Source: PLoS One. 2022 Jan 12;17(1):e0261151. doi: 10.1371/journal.pone.0261151 (PMC8754323; doi:10.1371/journal.pone.0261151)
Supplement: Attachment — Submitted filename: R2 - Revision Letter.docx [file pone.0261151.s003.docx]

Dear Dr. Proverbio,

We thank you for your feedback and the opportunity to revise our manuscript once more. Below you will find our detailed responses to your and the reviewer’s remaining comments. The original comments are presented in *Times New Roman (italic)* and our responses in Arial.

**Editor:**

*Besides dealing with the reviewer's comment, we would be grateful if you could consider and address, within the paper, the limitations commonly associated with the use of Amazon MTurk/Survey Monkey, namely, the non-naivety and trustworthiness of participants. Please also consider the opportunity of validating the results using another non-web-based sample. For further information, please see* [*http://www.annualreviews.org/doi/abs/10.1146/annurev-clinpsy-021815-093623*](http://www.annualreviews.org/doi/abs/10.1146/annurev-clinpsy-021815-093623) *and* [*http://journals.plos.org/plosone/article?id=10.1371/journal.pone.0057410#s15*](http://journals.plos.org/plosone/article?id=10.1371/journal.pone.0057410#s15)*. In alternative, please add a discussion dealing with these potential flaws*

We have added a paragraph to the Limitation section, considering the potential flaws of employing Amazon MTurk workers. In general, the evidence for low-quality data based on Amazon MTurk samples seems to be dependent on the specific research question and methods for ascertaining data quality (see Hauser et al., 2019 for a review). We now discuss the limited generalizability of our findings with regard to the employed sample. At the same time, we also emphasize the fact that we employed attention checks and detailed instructions to ascertain the quality of our study. The fact that we replicated the main findings from Schubert et al. (2018) using the same paradigm for videos also speaks to the validity of our findings.

We now write:

“The generalizability of the current findings hinges not only on the limited stimulus pool, but also on the employed sample. Amazon MTurk participants have been repeatedly criticized for providing low-quality data because they do not invest sufficient effort, are non-naive, and less trustworthy (e.g., Eyal et al., 2021). However, the evidence for these claims seems to be rather mixed and dependent on specific situations (Hauser et al., 2019). Hauser and colleagues (2019) highlight that high-quality data can be collected from MTurk, given specific research designs and an increased focus on attention checks. In the present study, we employed several attention checks and tried to make sure that participants understood the main task in order to avoid the possible pitfalls associated with MTurk participants. The fact that we replicated the main findings from a previous study using a similar paradigm (T. W. Schubert et al., 2018) also speaks to the validity of our results. Nevertheless, we acknowledge that conclusions beyond the present sample and also regarding possible cross-cultural differences in being moved responses (see Zickfeld et al., 2019) are necessarily limited.” (p. 37).

**R3:**

*The manuscript benefitted from the revision process. Many aspects and details have been corrected, added and/or clarified throughout the text. I am glad that the interpretation is now aligned more closely along the outcomes of the study. I also appreciate that (technical) aspects that could not be addressed or changed have been included into the limitations list.
I have however one remaining concern: the validity of the chills measures. As I already mentioned during the first round, the usual approach is to collect a dichotomous on-off-signal via button presses. My concern was that a continuous measure would inflate the occurrence of chills (which are short, peak-arousal phenomena). Unfortunately, this is exactly the case as we see now with the new additional average durations of 60 s. Most studies report much shorter instances of several chills/min/person (see for instance, Panksepp, 1995; Konecni et al. 2007; Benedek & Kaernbach 2011; Sumpf et al. 2015; Wassiliwizky, Koelsch et al. 2017; Wassiliwizky, Jacobsen et al. 2017; studies by Altenmüller’s group, etc.). I doubt that the authors measured the exactly same phenomenon as the studies they refer to.*

We understand the reviewer’s concern regarding the inflation of occurrence of chills, and believe that the important key lies in the definition of ‘chills’.

Consider the definitions summarized by de Fleurian & Pearce (2020, pp 2-3): “Frequently cited papers describe MECs as “a spreading gooseflesh, hair-on-end feeling that is common on the back of the neck and head and often moves down the spine” (Panksepp, 1995, p. 173), “a particularly intense, euphoric response to music [frequently accompanied] by an autonomic or psychophysiological component” (Blood & Zatorre, 2001, p. 11818), “intense emotional experiences involving sensations such as goose bumps or shivers down the spine” (Koelsch, 2010, p. 131), or “a pleasant tingling feeling associated with the flexing of hair follicles, resulting in gooseflesh (technically called piloerection) accompanied by a cold sensation, and sometimes producing a shiver” (Huron & Margulis, 2010, p. 591).”

While some of these definitions highlight that chills occur at *peak-arousal* (but not all), there is less mention of the fact that they are short-lived. In fact, some argue that intense occurrences of chills can be longer in duration than less intense experiences (Goldstein, 1980). In general, there seems to be no clear systematic evidence about the average duration of chills and this probably depends on the type of measurement and stimuli. Having a quick look at the studies the reviewer cited as evidence for chills instances being ‘much shorter’, reveals that Panksepp (1995), Benedek & Kaernbach (2011), and Sumpf et al., (2015) do not provide any specific evidence about the average duration of responses in their articles (at least, we couldn’t find them). For example, in Panksepp (1995) Study 6 occurrence of chills was recorded with participants having their hand raised while listening to music. In the results it seems that only the frequency of hands raised was recorded but not the duration. Similarly, Konecni et al. (2007) using the same methodology reported a mean duration of thrills at around 1-5s (the article reports durations as low as .60s, it seems unlikely that observers can record hand raises up to the millisecond precision though). Wassiliwizky, Jacobsen and colleagues (2017) found a mean duration of 33.8 seconds (with a maximum duration of 121s) for goosebump responses. Also looking at Figure 1 in Benedek and Kaernbach (2011) it seems that the depicted response lasts at least around 20s (in different intensities). Noteworthy, the mentioned studies focus on the phenomena of chills, goosebumps, or thrills. It is possible that these refer to different intensities or aspects of the same construct and therefore also produce different average durations.

It is also important to highlight that the average durations of 60.72s in the current study refer to different intensities. These were any intensities rated between 2-5 on the scale (with 2 referring to ‘A little chills’). Whether ratings below 3 or 4 would be covered by a dichotomous rating is questionable. Thereby, we might have included experiences that participants considered as chills to some degree that would not be considered if the rating was an all-or-nothing decision. There are of course advantages and disadvantages to both types of measurements. We mainly assume that subjective chills can occur in different intensities and this is possibly best captured by an ordinal response scale. If other researchers take the perspective that only responses that are high in intensity are considered ‘chills’, then a dichotomous measure is probably the superior choice. We now discuss this in the Limitation section.

Another way to interpret the current findings could be that chills or goosebumps come in consecutive waves that last only for a few seconds each, but these individual occurrences might be perceived as one combined episode. From this interpretation, chill episodes might be short lived, but they could be perceived as one longer instance if they occur in quick succession after another. In our rating it is unlikely that participants would indicate the absence of chills if there are breaks of only 1 or 2s between individual chill occurrences (which also reflects the usage of 3s time bins). The specific duration of chill responses might therefore also depend on the level of analysis and whether people are able to consciously differentiate between different chill episodes occurring in quick succession.

Finally, the reviewer mentions that they “doubt that the authors measured the exactly same phenomenon as the studies they refer to”. We cannot agree more, but would like to add that we would also doubt that the studies we are referring to are measuring the **exact** same phenomenon. As previously discussed, the definitions of chills have been quite diverse (see de Fleurian & Pearce, 2020). Some consider chills as the same phenomenon as goosebumps, others consider goosebumps an aspect of chills and so on. The different studies we reviewed sometimes refer to the phenomenon of chills, goosebumps, or thrills, which definitely have a lot in common, but are probably not the exact same phenomenon (see Maruskin et al., 2012). Therefore, we think that adequately defining the specific construct is crucial.

In general, we now discuss these issues in the Limitations section:

“Finally, in contrast to previous studies employing dichotomous ratings (e.g., Wassiliwizky, Jacobsen et al., 2017) we measured subjective chills using a continuous scale (based on Schubert et al., 2018). This was done in order to assess different intensities of chills or goosebumps. Importantly, we might therefore consider responses that would not be classified as chills responses with dichotomous rating paradigms. This is also obvious in the rather long average duration of chills ratings above the lowest scale point, suggesting that we not only covered peak-states but also lower intensities of subjective chills. Overall, we replicated associations between chills and different variables such as feeling moved, pointing to the validity of this measure. Notably, previous studies also differ in the reported average length of chills responses (Konečni et al., 2007; Wassiliwizky, Jacobsen et al., 2017), which is probably related to different methodologies and the exact operationalization of the phenomenon (i.e., as *chills, goosebumps,* or *thrills*). It is possible that for example *thrills* refer to a higher intensity response than *goosebumps* or that several short *chill* episodes occurring in quick succession are perceived as one longer response*.* A more standardized definition of the phenomenon of chills (see Maruskin et al., 2012) would be helpful in comparing different findings and evaluating the degree to which they investigate the same phenomenon.” (p. 39)

*Relatedly, I disagree with the statement in the manuscript: “some previous studies have assessed chills using a dichotomous measure probing whether participants experienced chills or not (p.18). Again, the dichotomous measurement is not an exception but the very standard. In the recent review on chills (which the authors refer to) by de Fleurian & Pearce (2020) it is said: “Most commonly, however, participants report chills by pressing on a button (Bannister, 2020b; Beier et al., 2020; Colver & El-Alayli, 2016; Egermann et al., 2011; Ferreri et al., 2019; Grewe et al., 2011; Grewe et al., 2009a; Grewe et al., 2007; Guhn et al., 2007; Laeng et al., 2016; Mas-Herrero et al., 2014; Mori & Iwanaga, 2014, 2015, 2017; Nagel et al., 2008; Rickard, 2004; Sachs et al., 2016; Salimpoor et al., 2011; Salimpoor et al., 2009; T. W. Schubert et al., 2018; Seibt et al., 2018; Starcke et al., 2019; Sutherland et al., 2009; Wassiliwizky, Koelsch, et al., 2017; Zickfeld, Schubert, Seibt, Blomster, et al., 2019). I think, this aspect should be addressed thoroughly in the Discussion part of the manuscript.*

Our intention was never to convey that assessing chills via a dichotomous scale is an exception. We have therefore slightly edited our statement to make this clear:

“Note, that previous studies have typically assessed chills using a dichotomous measure probing whether participants experienced chills or not (de Fleurian & Pearce, 2020).” (p. 18).

**R5**

*Reviewer #5: In the present paper, the author present the results of an online study that assessed people’s continuous ratings of musical excerpts with a particular focus on the relationship between ratings of “being moved”.

I understand that this paper has been revised already and I believe that it shows. Clearly, many supplementary analyses have been conducted to back up the main results. My review is based on the revised version alone, without regard to what aspects of the manuscript have been changed or to the responses to previous reviewers.

Overall, I find the paper well-written, the analyses rigorous, and the conclusions justified by the reported results. I particularly appreciate that all results are reported in terms of effect sizes and their confidence intervals. I therefore see no reason to object the publication of this paper given some rather minor changes, detailed below.*

*1. I think that the logic of pre-determining a musical piece as sadly or joyfully moving needs to be explained earlier on, otherwise, as a naïve reader, I would question the logic of two sections in the introduction:
a. Page 6, “The authors distinguish… “: I cannot follow the logic of tis sentence: Enjoying being moved (which thus needs to depend on being moved because it's a consequence) is only also moderated by being moved if one is sadly moved? How can a cause also moderate its effect?
b. Page 14: III. and IV.: I don't understand how this can be tested without double-dipping into the data*

The idea of the first statement is that according to Menninghaus and colleagues (2015) the link between feeling/perceiving sadness and enjoyment is mediated by being moved, but not the link between feeling/perceiving joy and enjoyment (or less so). Importantly, we are talking about a mediation here, not a moderation. These variants are called ‘sadly moving’ because they refer to stimuli or situations that are high on ratings of sadness and being moved, whereas the ‘joyfully moving’ variant is high on ratings of joy and being moved. We have now reformulated this sentence:

“They expect that being moved plays a more important role in the enjoyment of *sad* stimuli.” (p. 6)

Regarding the second point, we are not sure why the reviewer is referring to circular analysis here. The musical excerpts were characterized into sadly and joyfully moving excerpts prior to the study based on a pilot study, which can be found in the preregistration: <https://osf.io/76adr>. The only change persisted to the categorization of the original ‘neutral’ excerpt which was based on an error in the pilot study. Therefore, we don’t see how testing Hypothesis III and IV can be considered as ‘double-dipping’.

*2. P. 20: I find it unbalanced to suggest based on a single paper that time bins of three seconds constitute the temporal building block of perceptual-motor abilities. In fact, it seems very long to me given that much visual perceptual processing occurs at the time scale well below one second (face processing comes to mind especially). Most importantly for the current line of research, however, stable aesthetic ratings have been shown to occur within a second of stimulus presentation (e.g., Belfi et al., 2018; Brielmann, Vale, & Pelli, 2017). I think further justification – or alternatively the demonstration that the particular bin-width of aggregation does not matter as much – is needed.*

We added the acknowledgement that aesthetic responses can already occur at the 1s level. In fact, we had already provided evidence that the bin-width aggregation is of less importance by analyzing the data at the 1HZ resolution (Supplementary Table S8). This information is highlighted in Footnote 1.

*3. P. 26: Why only the mean and not also the peak? In time series ratings in the aesthetic domain, the peak has been identified as correlating highly with overall evaluations (e.g., Brielmann, Vale, & Pelli, 2017)*

We have explored the correlations between the empathic concern scores and peak ratings as suggested by the reviewer. Results are now presented in the Supplementary Material 2.3 and were nearly identical to the mean comparisons.

*4. Figure 6 (especially w regard to its mention on p. 26): to follow along with the interpretation of figure 6 in the text, it would be helpful to indicate which excerpts were joyfully and which ones sadly moving in figure 6 itself*

We have now revised Figure 6 and include an indication which excerpts were considered as joyfully and which were considered as sadly moving.

*5. P. 28 “strong initial rise within the first 30 seconds”: I think it should be also acknowledged that this might be at least partially dependent on the set starting point in this experiment, i.e., at the lowest end of the scale. So it's not clear whether this is an initial rise in actual responses to the music or just initial hesitation to move the displayed rating.*

We are not completely sure whether we understand the reviewer’s comment. We agree that the set starting point can influence this ‘initial rise’, though we do not quite see why an initial rise would be related to ‘hesitation to move the displayed rating’. If participants would hesitate to move the rating, we would probably not observe an initial rise.

Nevertheless, we added a short sentence to acknowledge the aspect that the starting point might influence our results:

“Notably, we cannot completely disentangle whether such a rise was caused by actual sudden changes in experiences or by a general need to change the rating because it always started at the lowest score.” (p. 28)

*6. P. 28, second paragraph: based on Figure 1 and the reported results, I cannot see how this statement is correct; cross-correlations appear just as high with perceived beauty and chills, and just as consistent*

Cross-correlations of feeling moved with sense of connection are technically the highest across all excerpts (*r* = .66), stronger than cross-correlations with chills (*r* = .63) or beauty (*r* = .60). These correlations are not significantly different from each other, though this is not something the statement suggested. In order to avoid misinterpretation of the statement, we have now added that “continuous ratings of feeling moved or touched had *one of* the highest cross-correlations overall with ratings of “feeling a sense of connection”.” (p. 28)

*7. P. 29 “Interestingly, these cross-correlations were consistent…”: There seems to be a missing argument or link between this and the previous sentence: Just because beauty and being moved are positively associated with one another in, overall, sad and joyful pieces does not imply anything about the association between sadness or joyfulness/happiness and beauty*

We have rearranged this paragraph in order to improve it. Previous studies have found that being moved mediated the relationship between sadness and beauty (but less so for joy and beauty), but in the current study we found positive correlations between feeling moved and beauty in both sad and joyful excerpts. In addition, the overall correlations between sadness/joy and beauty were different from previous studies:

“ Furthermore, in line with a previous study showing that movingness mediates the relationship between perceived sadness and beauty in music (Vuoskoski & Eerola, 2017), we found positive cross-correlations between ratings of feeling moved or touched and perceived beauty. Interestingly, these cross-correlations were consistent across both sadly and joyfully moving excerpts. Previous research has associated perceived beauty with perceived sadness and only to a lower degree with perceived happiness (*r* = .59 vs. *r* = .16; Eerola & Vuoskoski, 2011), while in the current study we found overall weaker associations between perceived sadness and beauty than between perceived joy and beauty (*r* = .12 vs. *r* = .54).” (pp. 29-30)
